# Supplementary material for: Single-molecule imaging reveals multiple pathways for the recruitment of translesion polymerases after DNA damage
Source: Nat Commun. 2017 Dec 18;8:2170. doi: 10.1038/s41467-017-02333-2 (PMC5735139; doi:10.1038/s41467-017-02333-2)
Supplement: Supplementary file 1 — Supplementary Information [file 41467_2017_2333_MOESM1_ESM.pdf]

Supplementary Information

SUPPLEMENTARY FIGURES

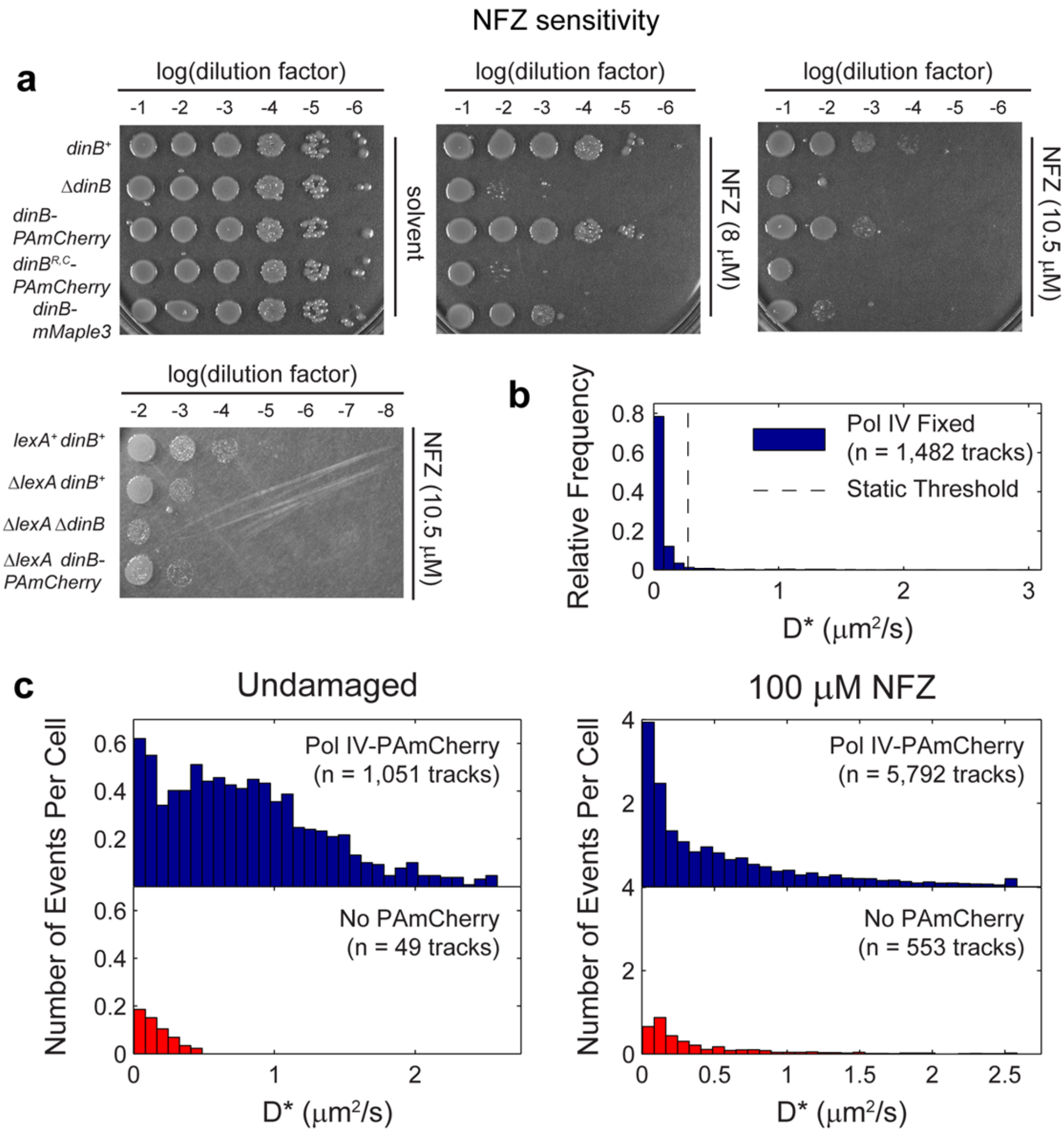

Supplementary Figure 1. Additional NFZ sensitivity assay data and control experiments for Pol IV-PAmCherry diffusion measurements. (a) Top panels: Serial 10-fold dilutions of *E. coli* strains in the wildtype MG1655 background grown on LB agar plates without (left) and with nitrofurazone (NFZ) added at 8 μM (middle) or 10.5 μM (right) concentration, revealing only a modest TLS defect for the fusions. Bottom panel: Serial 10-fold dilutions of the *E. coli* strains

from Fig. 1b grown on LB agar plates with NFZ added at 10.5 μM concentration. (b) Distribution of the apparent diffusion coefficient (*D**) for Pol IV-PAmCherry in cells fixed with formaldehyde. The dashed line indicates the threshold *D** value for bound molecules ($D^* <$ 0.275 $\mu m^2\ s^{-1}$) calculated from this distribution. (c) The *D** distributions for undamaged (left) and 100 μM NFZ-treated (right) cells plotted on a per-cell basis for cells with (top panels) and without (bottom panels) PAmCherry fused to Pol IV. Data in the top panels are replotted from Fig. 1d to enable comparison.

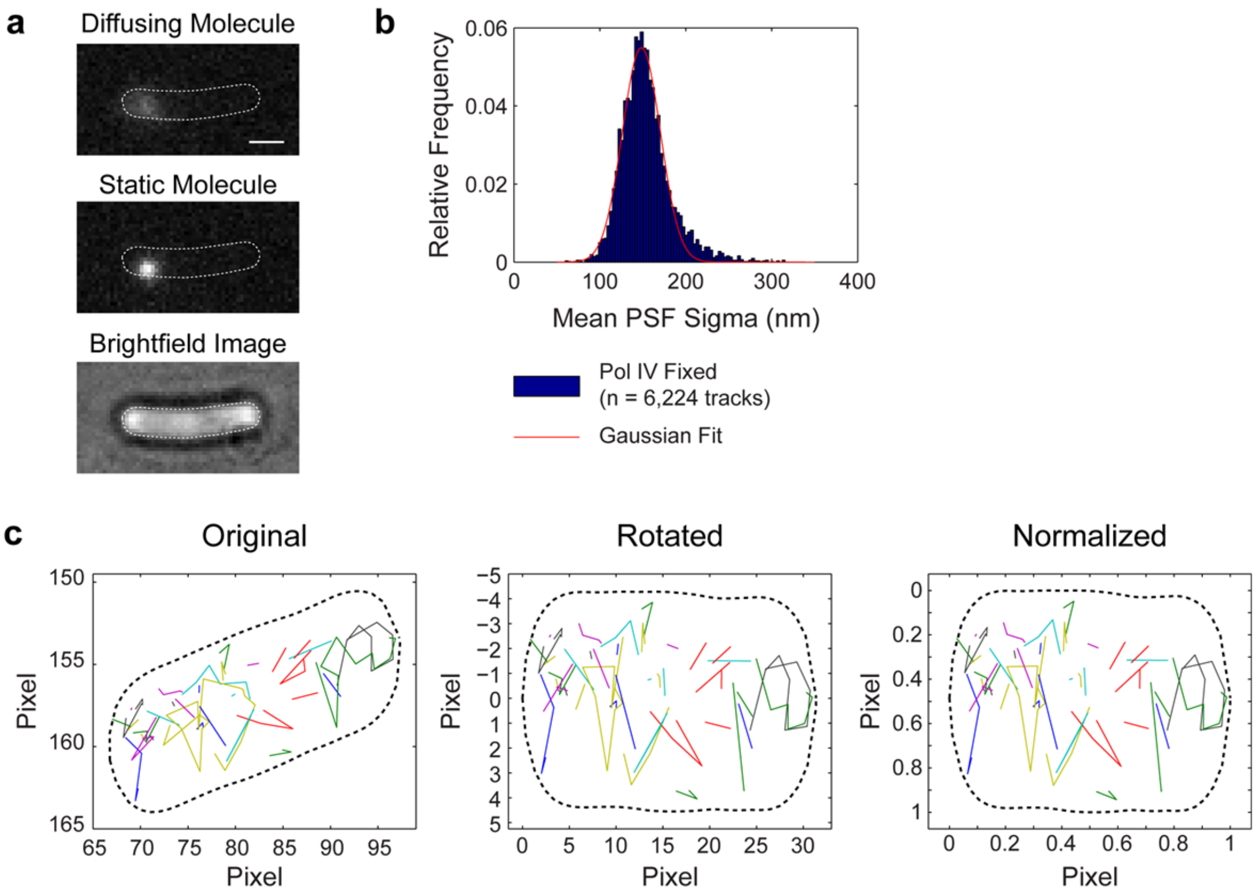

Supplementary Figure 2. Control experiments and schematics for static Pol IV-PAmCherry cellular localization. (a) Representative fluorescence micrographs of diffusing (top) and static (middle) Pol IV-PAmCherry PSFs recorded with 250 ms integration times, and the corresponding brightfield image, with overlays of the cell outlines. (Scale bar: 1 µm.) (b) Distribution of the mean track PSF width in cells fixed with formaldehyde, and the Gaussian fit to the distribution (red line) with a mean ± standard deviation of 148.3 ± 31.4 nm. (c) Schematic of the cell rotation and normalization procedure. Left panel: original cell outline and detected tracks. Middle panel: rotated cell outline and tracks. Right panel: rotated and normalized cell outline and tracks.

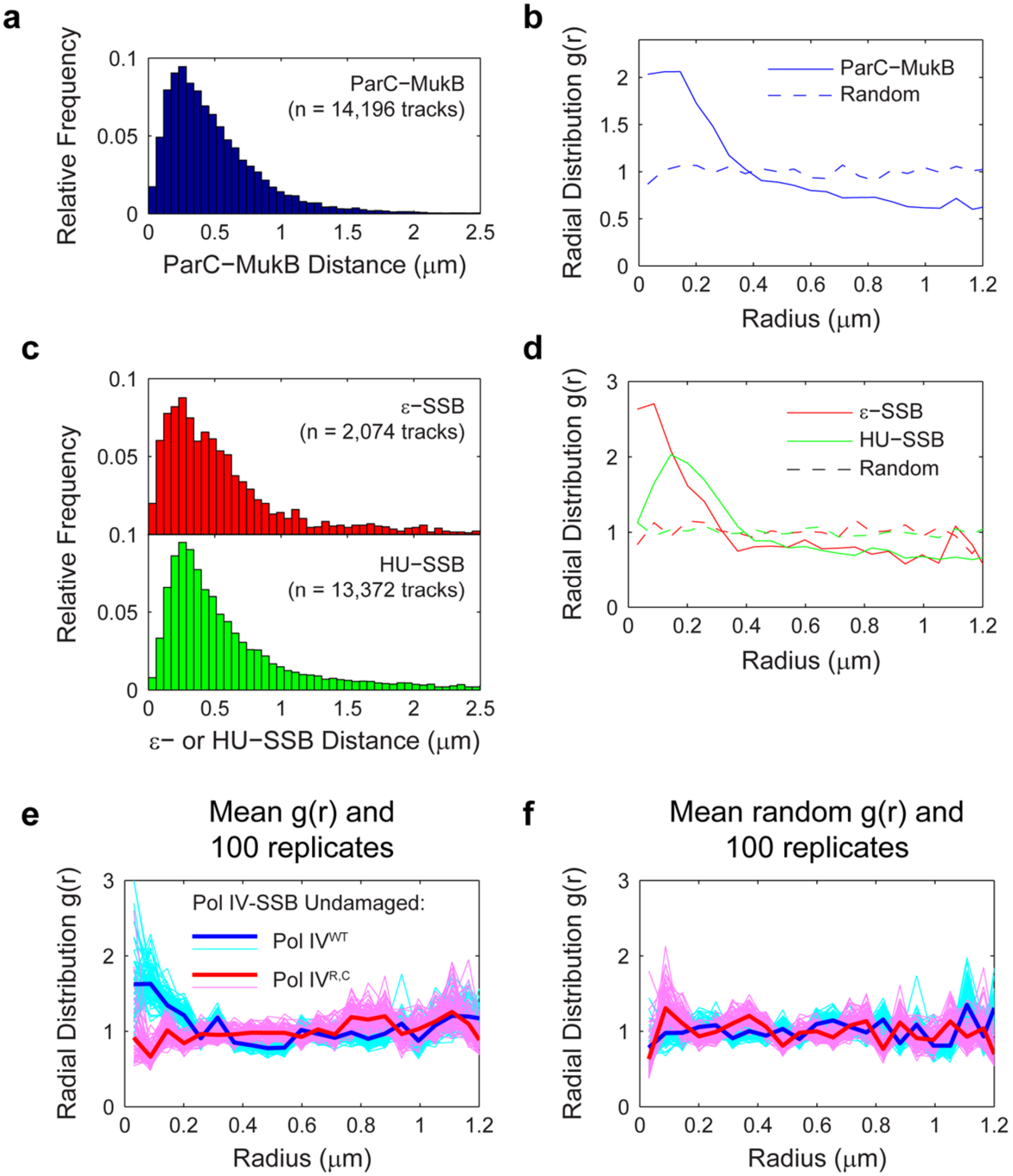

Supplementary Figure 3. Colocalization control experiments. Distributions of the mean distance between each static ParC-PAmCherry track and the nearest MukB-mYPet focus in undamaged cells (a) and the corresponding radial distribution function *g(r)* (b). Distributions of

the mean distance between each static ε- or HU-PAmCherry track and the nearest SSB-mYPet focus in undamaged cells (c) and the corresponding radial distribution functions *g(r)* (d). Also shown in (b) and (d) are random *g(r)* functions for each dataset. (e) Pol IV-SSB radial distribution functions *g(r)* for Pol IV^{WT} and the Pol $IV^{R,C}$ mutant in undamaged cells. Shown are the 100 *g(r)* curves for each dataset (thin lines) and the mean *g(r)* curves (thick lines). (f) As in (e), but for the random *g(r)* curves. The mean *g(r)* curves in (e) and (f) are replotted from Fig. 3b.

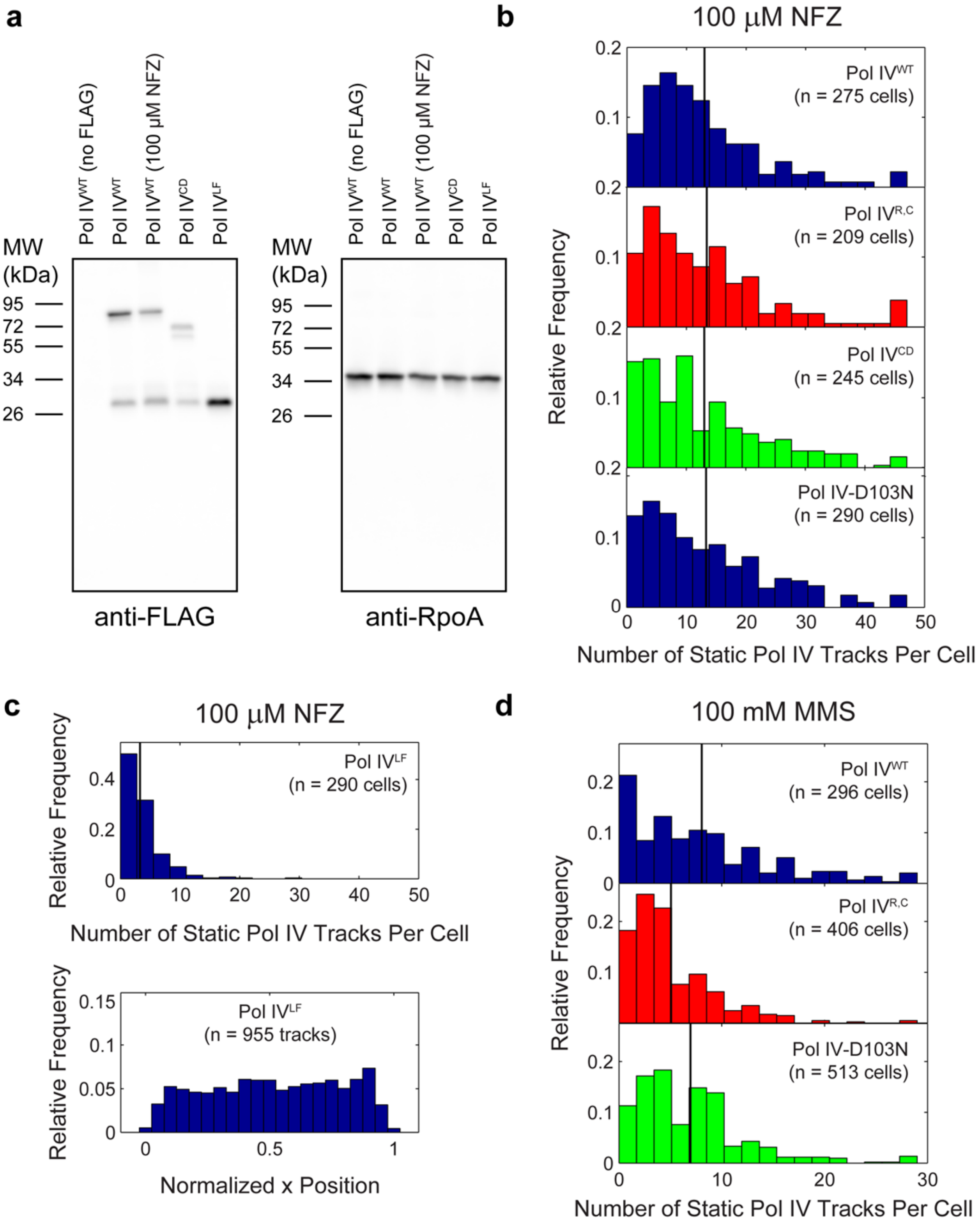

Supplementary Figure 4. Western blot analysis of FLAG-tagged Pol IV mutants and effect of DNA damage on Pol IV mutants. (a) Western blot analysis of strains containing Pol IV-

PAmCherry (lane 1), Pol IV-PAmCherry-FLAG (lane 2: undamaged; lane 3: treated with 100 μM NFZ), Pol IV^{CD}-PAmCherry-FLAG (lane 4), and Pol IV^{LF}-PAmCherry-FLAG (lane 5) constructs. Blots were probed with an anti-FLAG antibody to look at Pol IV expression (left) or an anti-RpoA antibody as a loading control (right). A small cleavage product is visible for all FLAG-tagged strains in the anti-FLAG blot, which likely corresponds to the cleaved PAmCherry-FLAG fragment. This product would not be observed in imaging of statically bound molecules (see Supplementary Fig. 5a,b). For the Pol IV^{LF}-PAmCherry-FLAG construct, this product is the only species present. For uncropped Western blot images, see Supplementary Fig. 18. (b) Distributions of the number of static Pol IV-PAmCherry tracks per cell in cells treated with 100 μM NFZ for Pol IV mutants. The Pol IV^{WT} data in the top panel are repeated from Fig. 4a to enable comparison. The mean of each distribution is indicated by a solid line. (c) Top panel: Distributions of the number of static tracks per cell for the Pol IV^{LF} construct in cells treated with 100 μM NFZ. The mean of the distribution is indicated by a solid line. Bottom panel: Long-axis cellular localization of the Pol IV^{LF} construct in cells treated with 100 μM NFZ. (d) Distributions of the number of static Pol IV-PAmCherry tracks per cell in cells treated with 100 mM MMS for Pol IV^{WT} and the Pol $IV^{R,C}$ and Pol IV-D103N mutants. The Pol IV^{WT} data in the top panel are replotted from Fig. 4a to enable comparison. The mean of each distribution is indicated by a solid line.

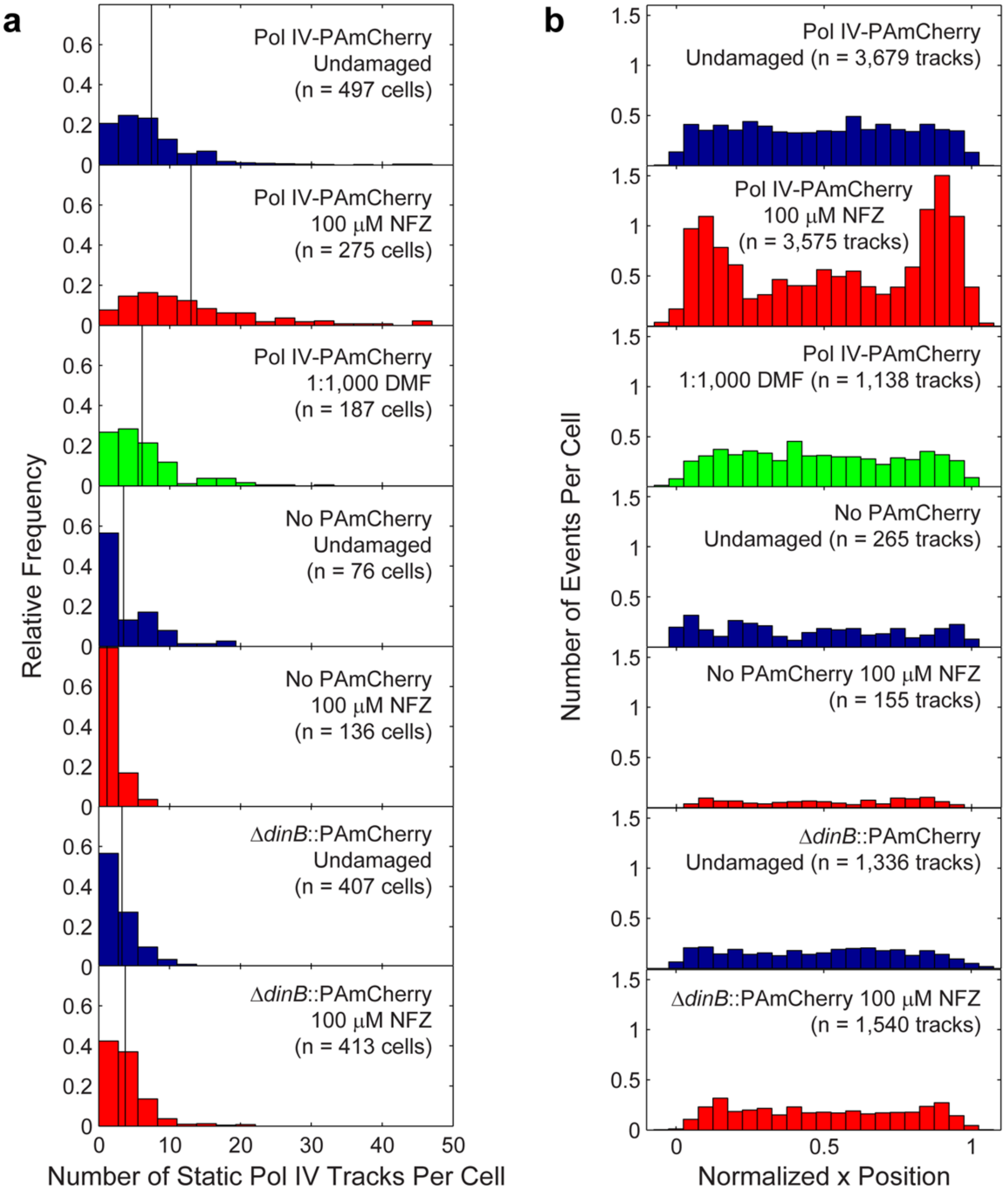

Supplementary Figure 5. Control experiments for Pol IV-PAmCherry tracks and cellular localization in undamaged and NFZ-treated cells. (a) Distributions of the number of static tracks per cell in undamaged cells and cells treated with 100 μM NFZ or a 1:1,000 dilution of dimethylformamide (DMF) as a solvent control. Pol IV-PAmCherry: strain containing both Pol

IV-PAmCherry and SSB-mYPet fusions. No PAmCherry: strain containing an SSB-mYPet fusion only. Δ*dinB*::PAmCherry: strain containing the *dinB* gene replaced by PAmCherry and an SSB-mYPet fusion. The Pol IV-PAmCherry data in the top two panels are repeated from Fig. 4a to enable comparison. The mean of each distribution is indicated by a solid line. Mean number of static tracks ± s.e.m. and p-values: Pol IV-PAmCherry DMF (6.1 ± 0.4, $p \ll 10^{-5}$ vs. Pol IV-PAmCherry 100 μM NFZ), No PAmCherry undamaged (3.5 ± 0.5, $p \ll 10^{-5}$ vs. Pol IV-PAmCherry undamaged), No PAmCherry 100 μM NFZ (1.1 ± 0.1, $p \ll 10^{-5}$ vs. Pol IV-PAmCherry 100 μM NFZ), Δ*dinB*::PAmCherry undamaged (3.3 ± 0.2, $p \ll 10^{-5}$ vs. Pol IV-PAmCherry undamaged), Δ*dinB*::PAmCherry 100 μM NFZ (3.7 ± 0.2, $p \ll 10^{-5}$ vs. Pol IV-PAmCherry 100 μM NFZ). (b) Long-axis cellular localization of static tracks in undamaged cells and cells treated with 100 μM NFZ plotted on a per-cell basis. The Pol IV-PAmCherry data in the top two panels are replotted from Fig. 2a,c to enable comparison.

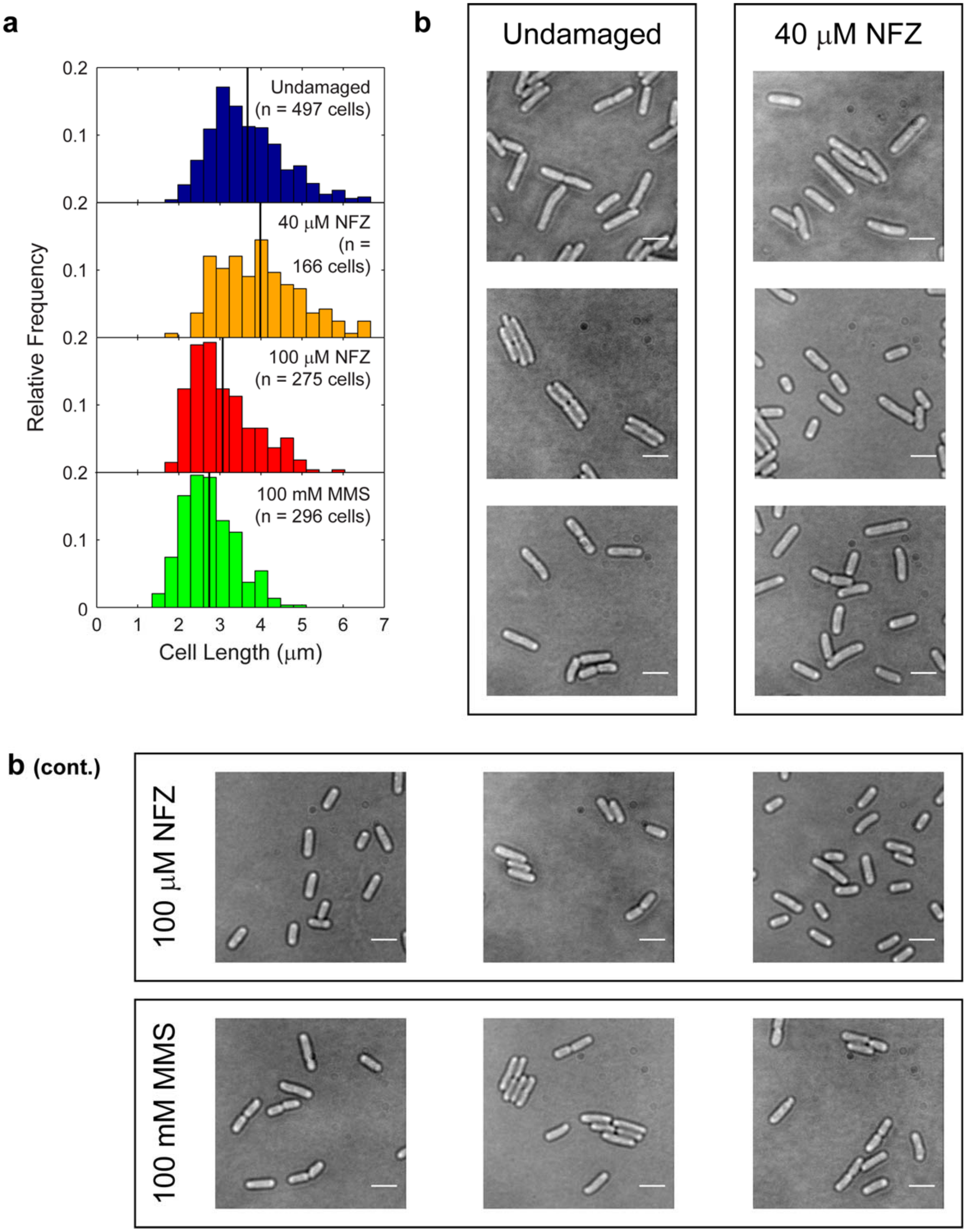

Supplementary Figure 6. Cell length distributions and brightfield micrographs of undamaged, NFZ-treated, and MMS-treated cells. (a) Distributions of the cell length in

undamaged cells and cells treated with 40 µM NFZ, 100 µM NFZ, or 100 mM MMS. The mean of each distribution is indicated by a solid line. Mean cell length (µm) ± s.e.m: Undamaged (3.67 ± 0.04), 40 µM NFZ (3.98 ± 0.08), 100 µM NFZ (3.07 ± 0.05), 100 mM MMS (2.74 ± 0.04). (b) Representative brightfield micrographs of three separate, randomly selected fields of view for the conditions in (a). (Scale bars: 3 µm.)

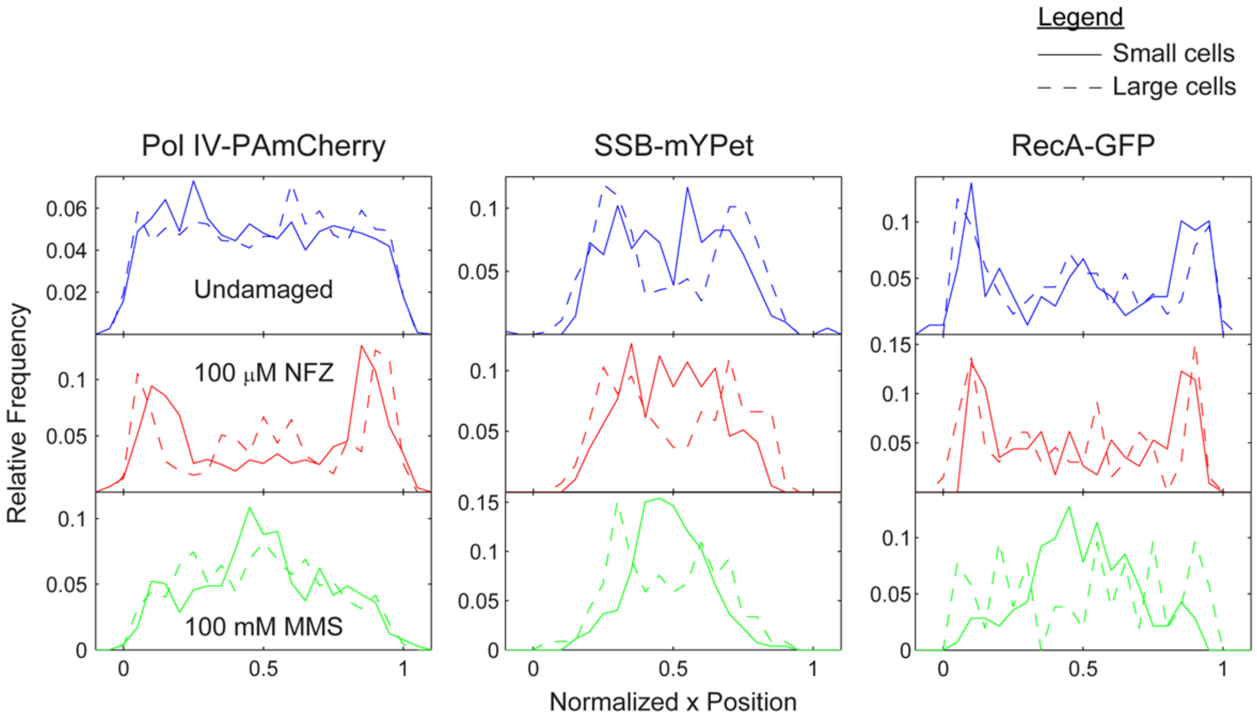

Supplementary Figure 7. The effect of cell size on the cellular localization distributions of Pol IV, SSB, and RecA. Long-axis cellular localization distributions in undamaged (top panels), 100 μM NFZ-treated (middle panels), and 100 mM MMS-treated (bottom panels) cells for Pol IV-PAmCherry (left panels), SSB-mYPet (middle panels), and RecA-GFP (right panels) for small (solid lines) and large (dashed lines) cells. A cell length threshold of 3.2 μm was used to differentiate between small and large cells. The results demonstrate that the characteristic localization patterns of Pol IV and RecA under different treatment conditions do not reflect differences in average cell size, whereas SSB foci are located at the quarter cell position in large cells and closer to midcell in small cells under all treatment conditions. Number of cells: Pol IV undamaged (186 small, 311 large), Pol IV 100 μM NFZ (179 small, 96 large), Pol IV 100 mM MMS (231 small, 65 large); SSB undamaged (168 small, 283 large), SSB 100 μM NFZ (230 small, 110 large), SSB 100 mM MMS (231 small, 65 large); RecA undamaged (107 small, 99 large), RecA 100 μM NFZ (137 small, 58 large), RecA 100 mM MMS (218 small, 56 large).

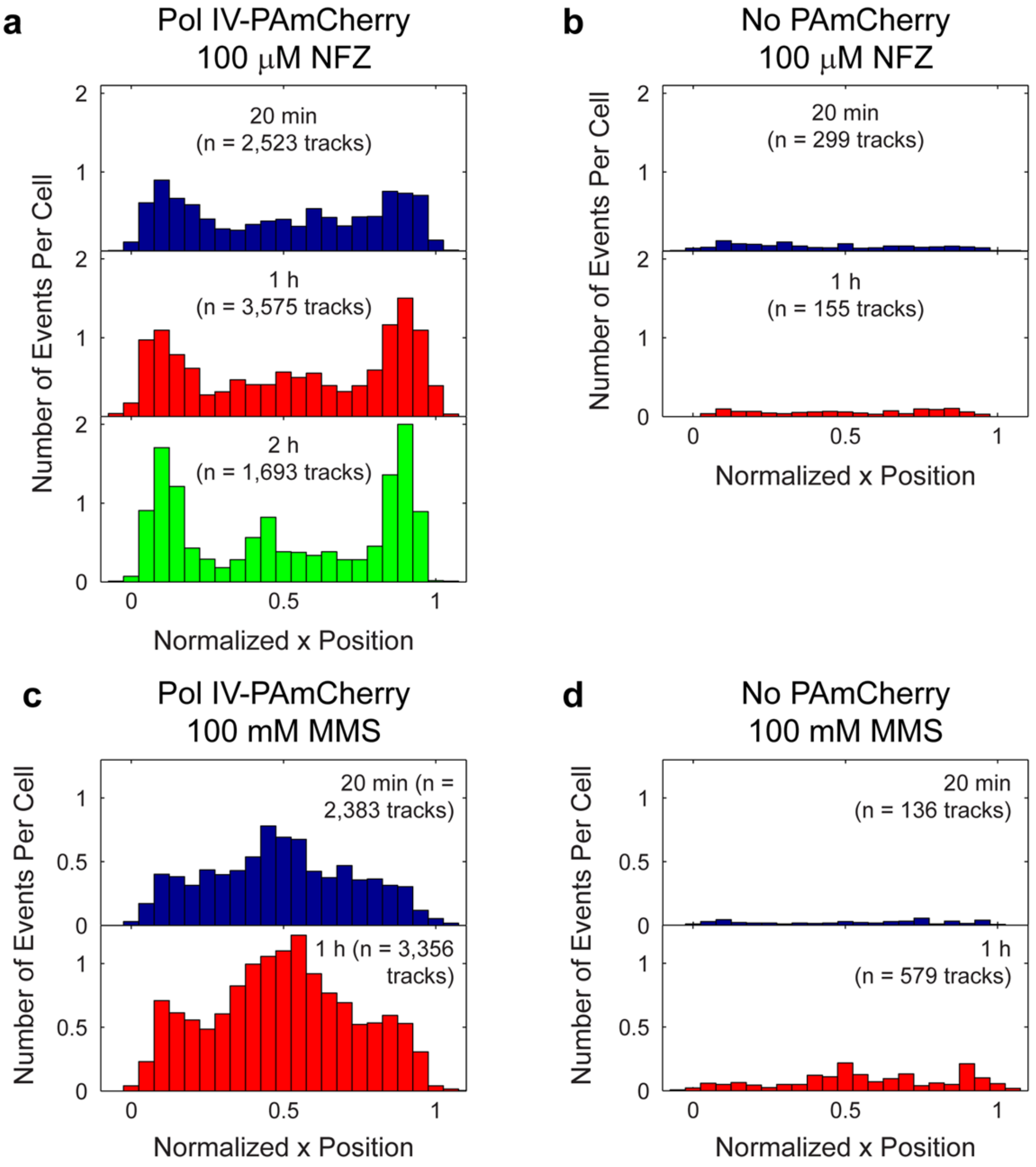

Supplementary Figure 8 Effect of incubation time on Pol IV cellular localization in NFZ- and MMS-treated cells. Long-axis cellular localization of static tracks in cells treated with 100 μM NFZ for 20 min, 1 h, or 2 h (a and b) or 100 mM MMS for 20 min or 1 h (c and d) plotted on a per-cell basis. The data in the middle panel of (a), the bottom panel of (b), and the top panel of (c) are replotted from Fig. 2c, Supplementary Fig. 5b, and Fig. 2e respectively to enable comparison. Strain legend as in Supplementary Fig. 5.

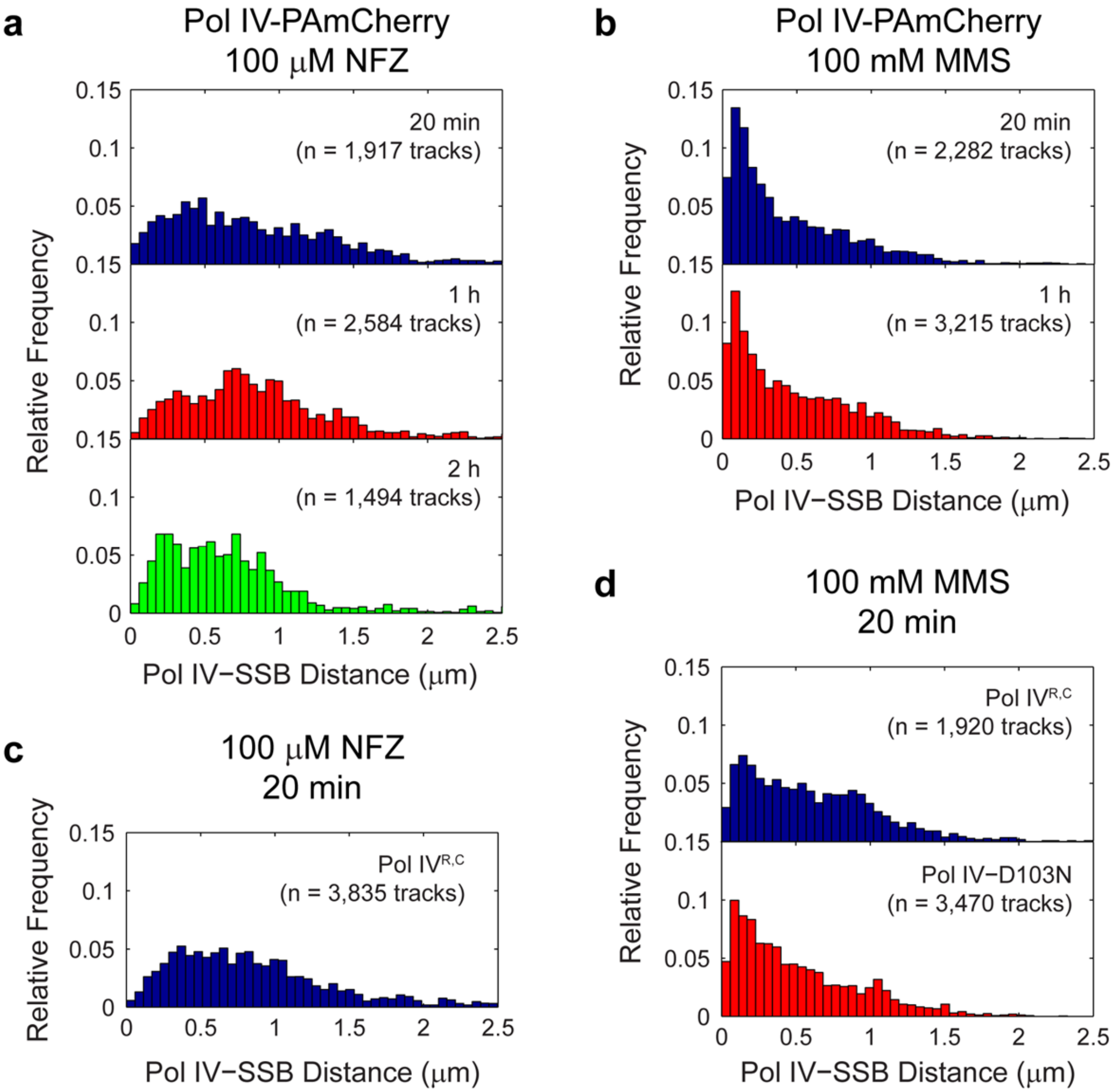

Supplementary Figure 9. Effect of incubation time on Pol IV-SSB colocalization. (a) Distributions of the mean distance between each static Pol IV track and the nearest SSB focus in cells treated with 100 μM NFZ for 20 min, 1 h, or 2 h (a) or 100 mM MMS for 20 min or 1 h (b). The 100 μM NFZ 1 h and 100 mM MMS 20 min data are replotted from Fig. 3 to enable comparison. (c) Distribution of the mean distance between each static Pol IV track and the nearest SSB focus for the Pol IV^{R,C} mutant in cells treated with 100 μM NFZ (d) or for the Pol IV^{R,C} and Pol IV-D103N mutants in cells treated with 100 mM MMS.

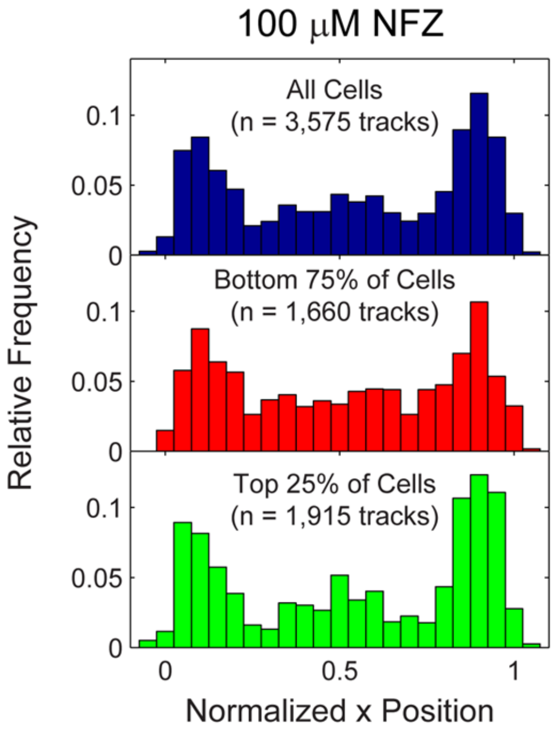

Supplementary Figure 10. Cellular localization profiles of Pol IV is largely independent of localization number in NFZ-treated cells. Long-axis cellular localization of Pol IV-PAmCherry in cells treated with 100 μM NFZ divided into two populations based on the number of static tracks per cell. The top panel shows localization across all cells, the middle panel shows localization across cells in the bottom 75% based on number of tracks, and the bottom panel shows localization across cells in the top 25% based on number of tracks. The data in the top panel are replotted from Fig. 2c to enable comparison.

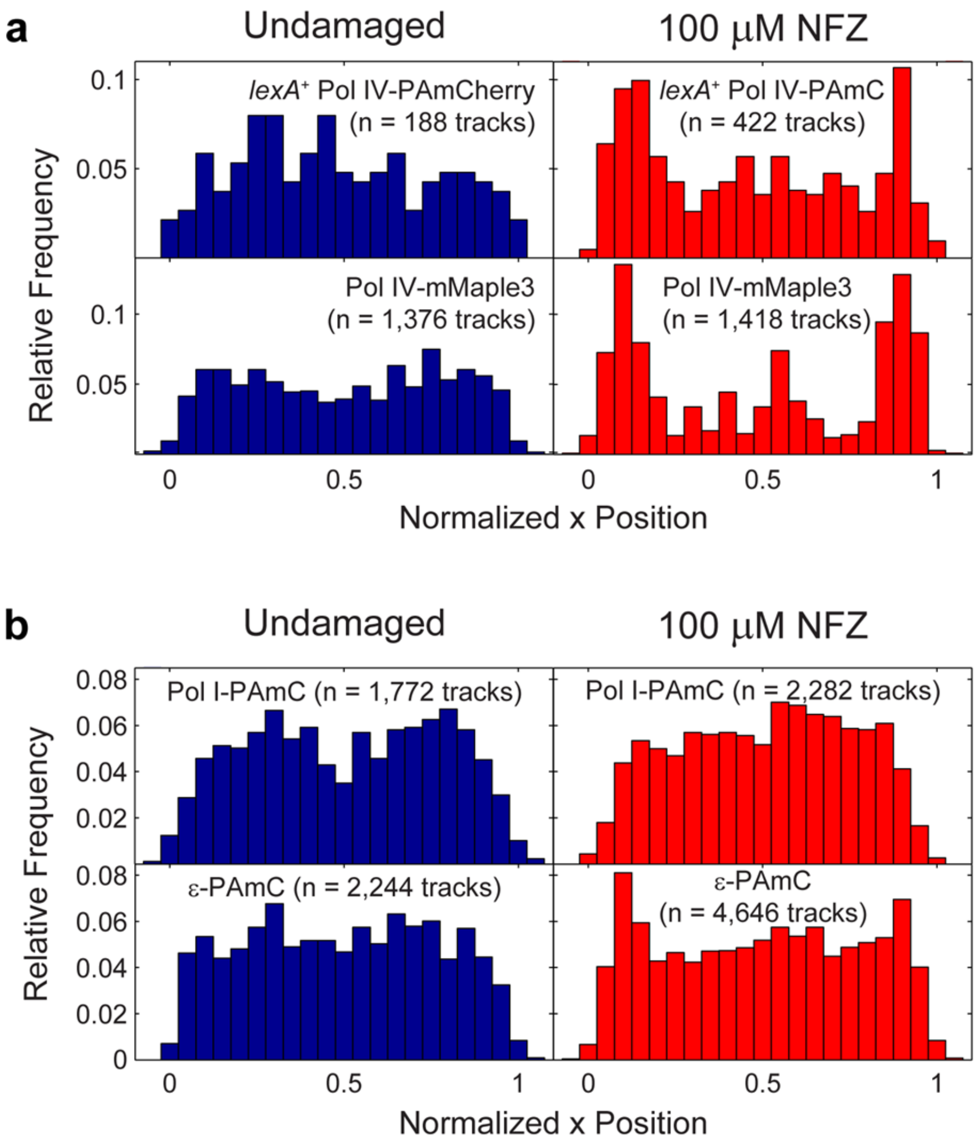

Supplementary Figure 11. Control experiments for Pol IV cellular localization in undamaged, NFZ-treated, and MMS-treated cells and cellular localization of other replication factors. (a) Long-axis cellular localization distributions in undamaged (left) and 100 μM NFZ-treated (right) cells for a Pol IV-PAmCherry fusion in the *lexA*$^+$ background (top panels) and a Pol IV-mMaple3 fusion in the *ΔlexA* background (bottom panels). (b) Long-axis cellular localization of Pol I-PAmCherry (top panels) and ε-PAmCherry (bottom panels) in undamaged cells (left) and cells treated with 100 μM NFZ (right).

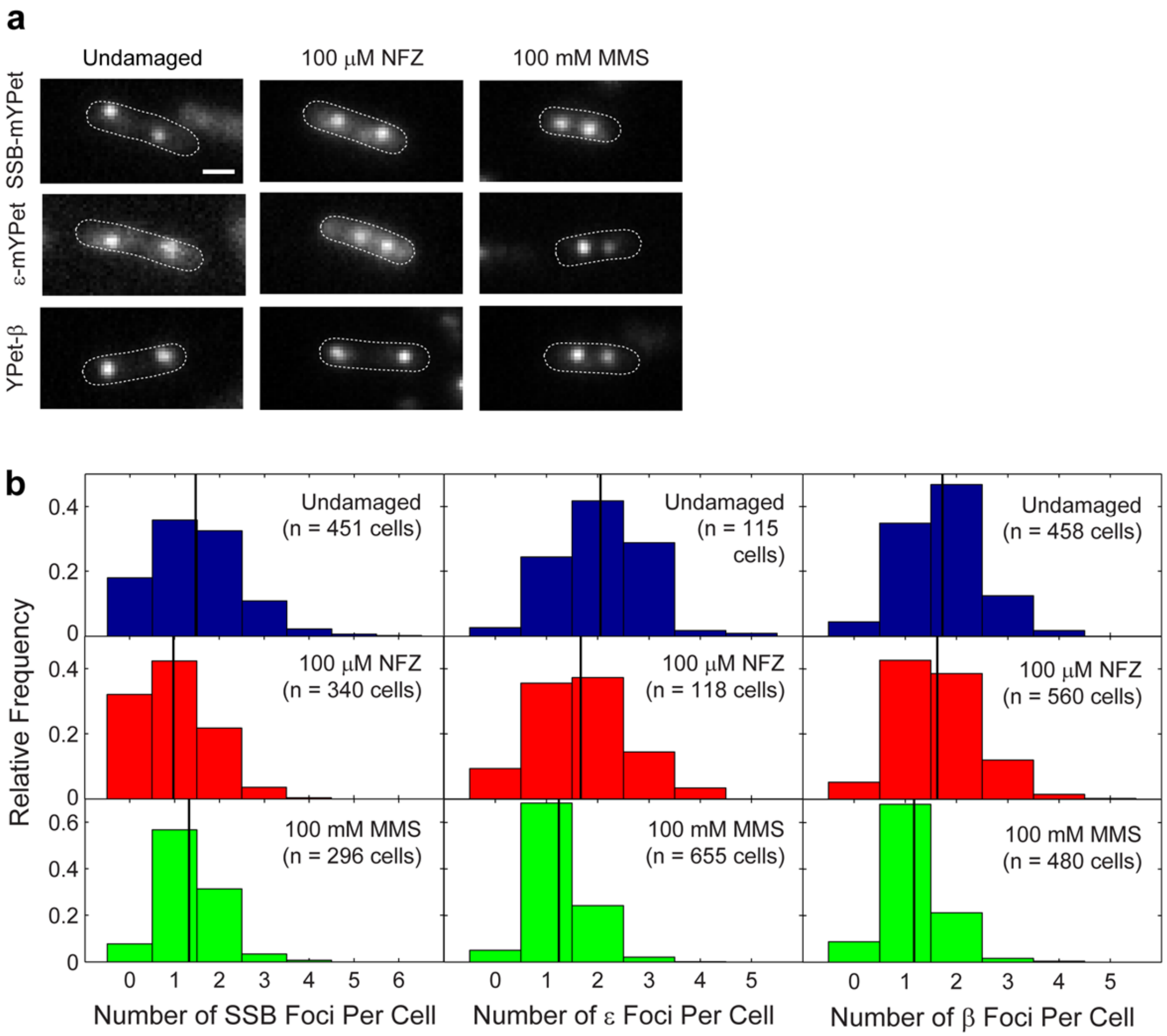

Supplementary Figure 12. Effect of NFZ and MMS treatment on the replisome. (a) Micrographs of SSB-mYPet (top), ε-mYPet (middle), and YPet-DnaN (bottom) foci in undamaged (left), 100 μM NFZ-treated (middle), and 100 mM MMS-treated (right) cells, with overlays of the cell outlines. For comparison, cells with two foci are shown under all conditions. (Scale bar: 1 μm). (b) Distributions of the number of SSB-mYPet (left), ε-mYPet (middle), and YPet-β (right) foci per cell in undamaged (top), 100 μM NFZ-treated (middle), and 100 mM MMS-treated (bottom) cells. The mean of each distribution is indicated by a solid line.

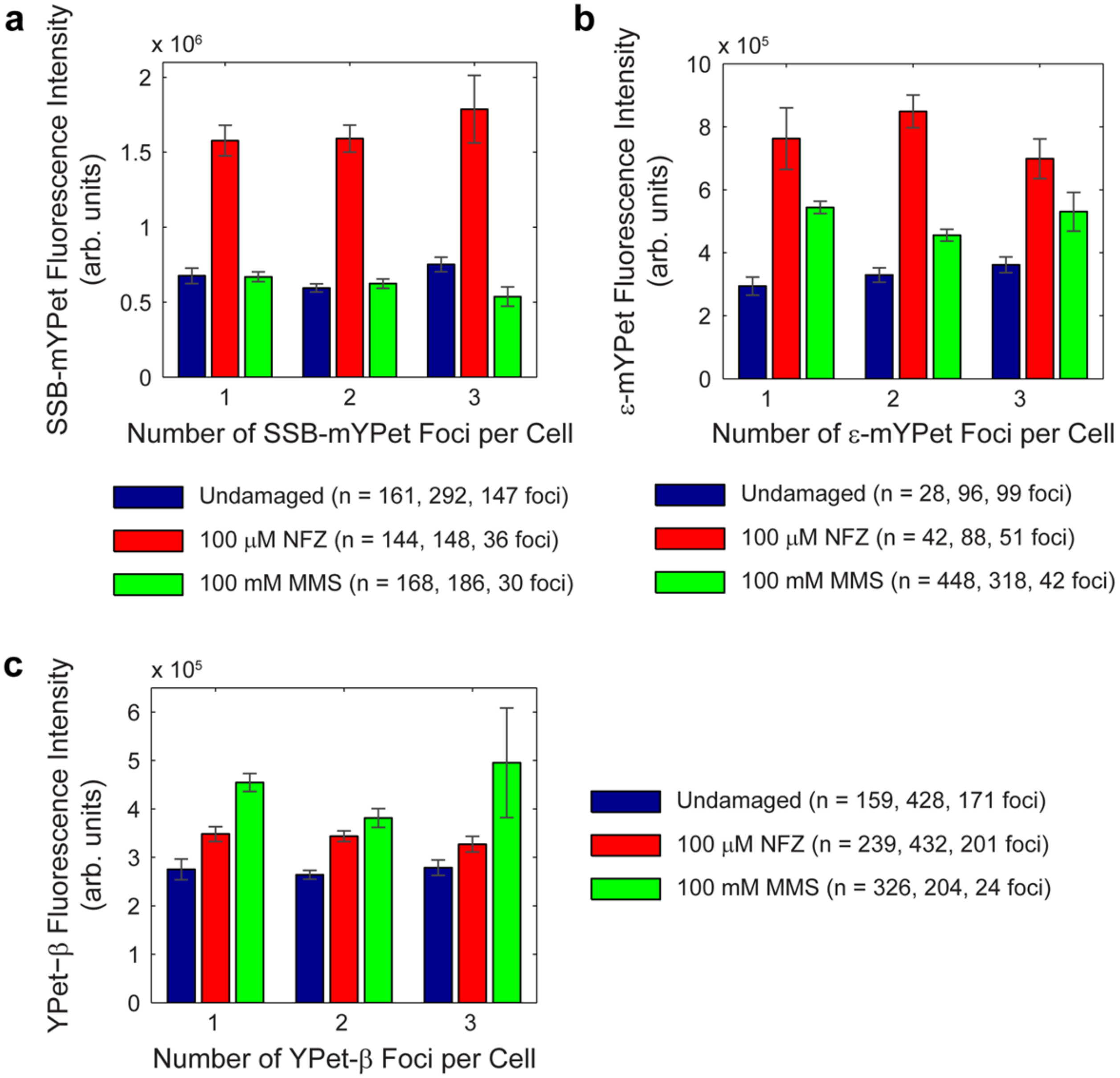

Supplementary Figure 13. Effect of NFZ and MMS treatment on the intensity of replisome foci. Integrated fluorescence intensity of (a) SSB-mYPet, (b) ε-mYPet, and (c) YPet-β foci in undamaged, NFZ-treated, and MMS-treated cells (left to right) as a function of the number of foci in the cell. (Error bars: standard error of the mean.) All differences between undamaged and NFZ- or MMS-treated cells are statistically significant at the $p < 0.05$ level with the exception of the comparison between undamaged and MMS-treated cells containing 2 SSB-mYPet foci or 3 YPet-β foci.

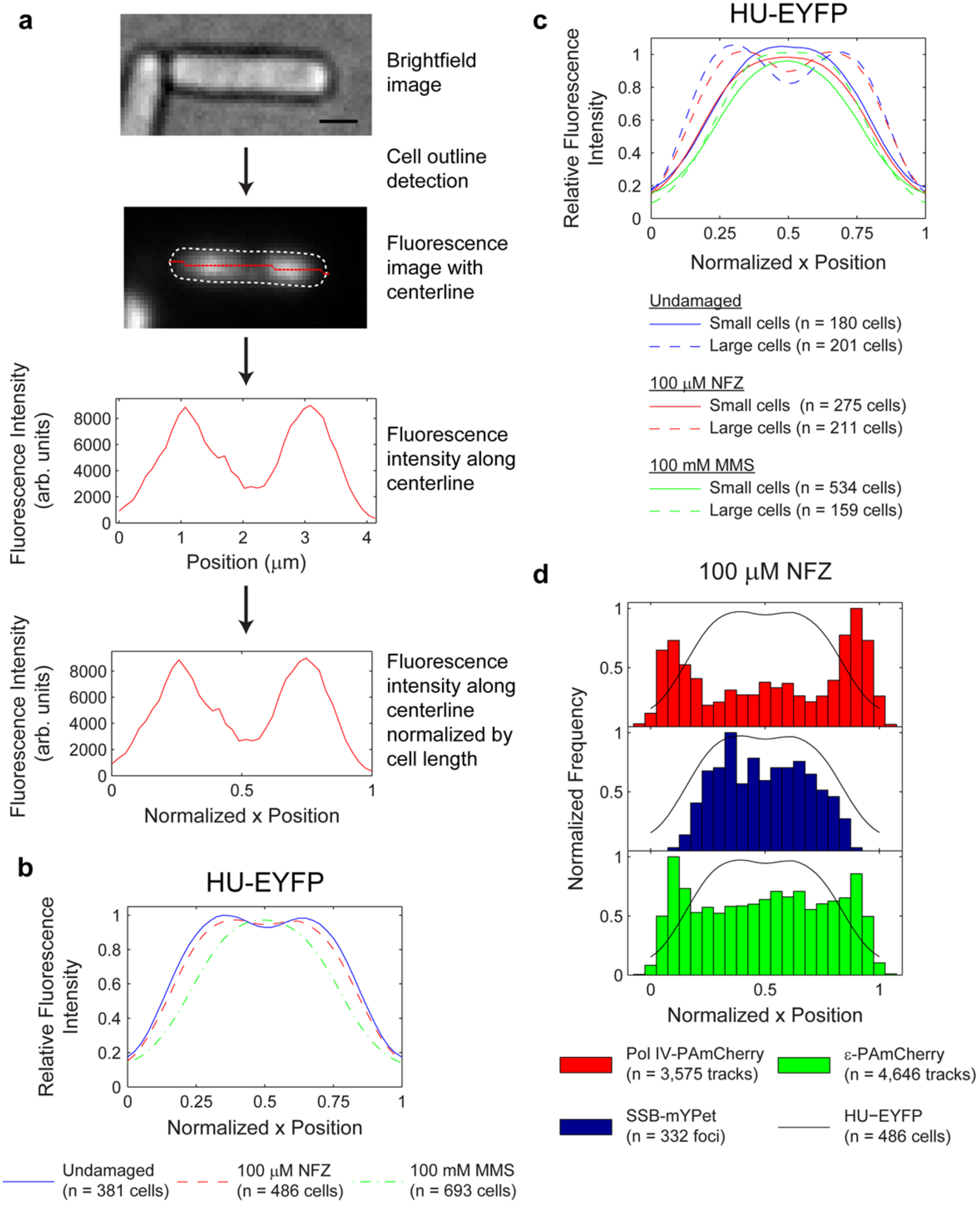

Supplementary Figure 14. Effect of NFZ and MMS treatment on the nucleoid. (a) Schematic of the HU-EYFP intensity analysis procedure. Top panel: representative brightfield micrograph. Top middle panel: fluorescence micrograph with an overlay of the cell outline

(white dashed line) and the long-axis centerline (red line). Bottom middle panel: fluorescence intensity along the cell centerline. Bottom panel: fluorescence intensity plotted against the normalized cell length to generate a normalized intensity profile for averaging. (Scale bar: 1 µm.) (b) Characterization of the nucleoid extent by mean HU-EYFP intensity profiles along the cell long axis centerline for undamaged (blue solid line), 100 µM NFZ-treated (red dashed line), and 100 mM MMS-treated (green dash-dotted line) cells. All profiles are normalized to the maximum intensity in undamaged cells. (c) Comparison of the average HU-EYFP intensity profiles in undamaged cells (blue) and cells treated with 100 µM NFZ (red) or 100 mM MMS (green) for small (solid lines) and large (dashed lines) cells. A cell length threshold of 3.2 µm was used to differentiate between small and large cells. The HU-EYFP intensity profile is peaked at midcell for both small and large MMS-treated cells. (d) The average HU-EYFP intensity profile in cells treated with 100 µM NFZ and the long-axis cellular localization distributions for Pol IV-PAmCherry (top), SSB-mYPet (middle), and ε-PAmCherry (bottom) under the same conditions. The HU-EYFP intensity profile is repeated from (b). The cellular localization data in the top and middle panels are repeated from Fig. 2c,d and the data in the bottom panel are repeated from Supplementary Fig. 11b to enable comparison.

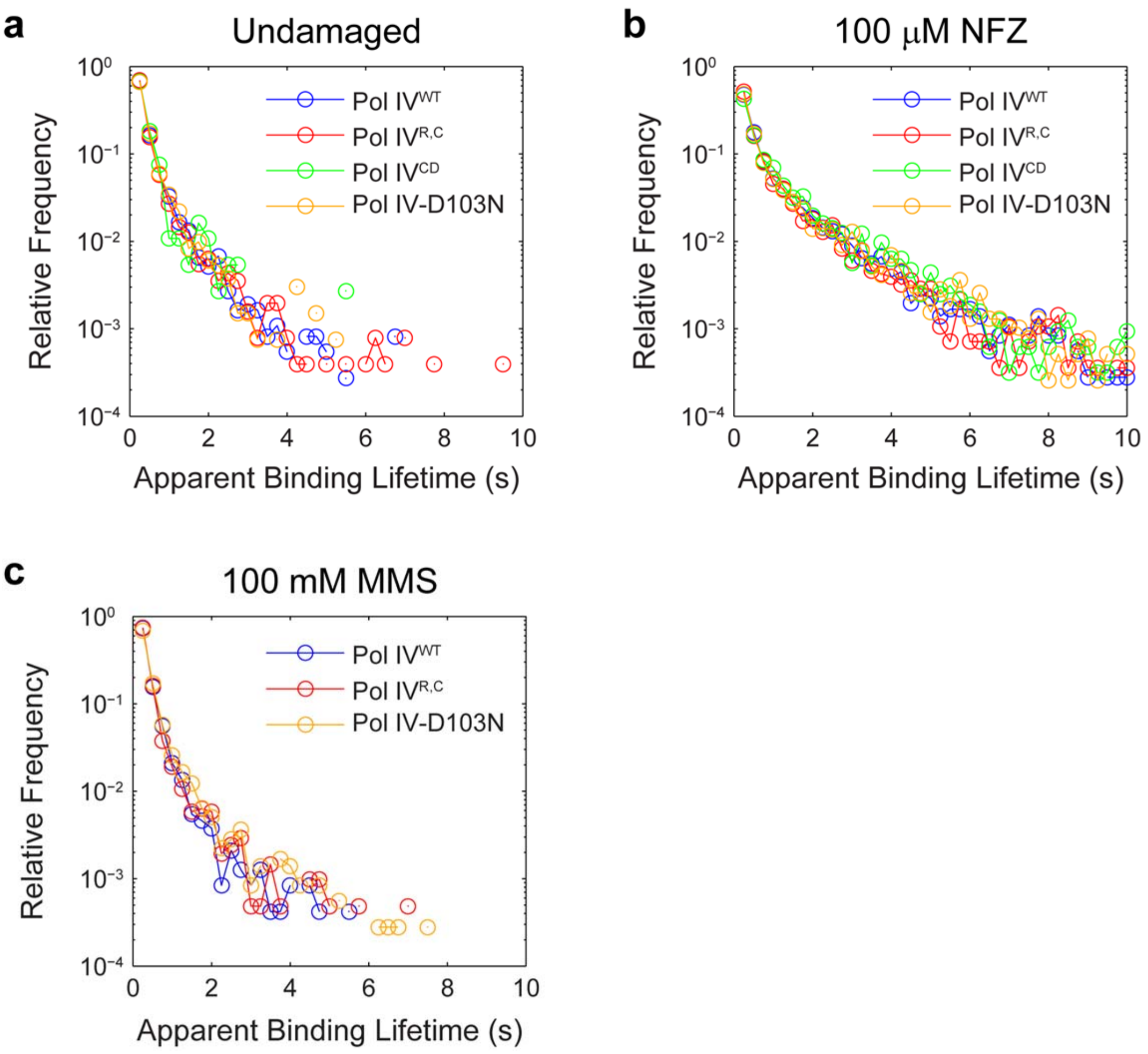

Supplementary Figure 15. Binding lifetime of Pol IV mutants in undamaged cells and cells treated with NFZ and MMS. (b) Distributions of the apparent Pol IV-PAmCherry binding lifetime in undamaged cells (a) and cells treated with 100 μM NFZ (b) or 100 mM MMS (c) for Pol IV^{WT} and the Pol IV^{R,C}, Pol IV^{CD} (undamaged and NFZ only), and Pol IV-D103N mutants. Number of tracks: undamaged (371 – 3,679), 100 μM NFZ (2,803 – 3,869), 100 mM MMS (2,057 – 3,570). Percentage change in mean binding lifetime relative to Pol IV^{WT} and p-values: Pol IV^{R,C} 100 μM NFZ (– 2%, $0.1 < p < 0.5$), Pol IV^{CD} 100 μM NFZ (+ 19%, $p < 10^{-5}$), Pol IV-D103N 100 mM MMS (+ 13%, $p < 10^{-3}$). All other differences NS relative to Pol IV^{WT}.

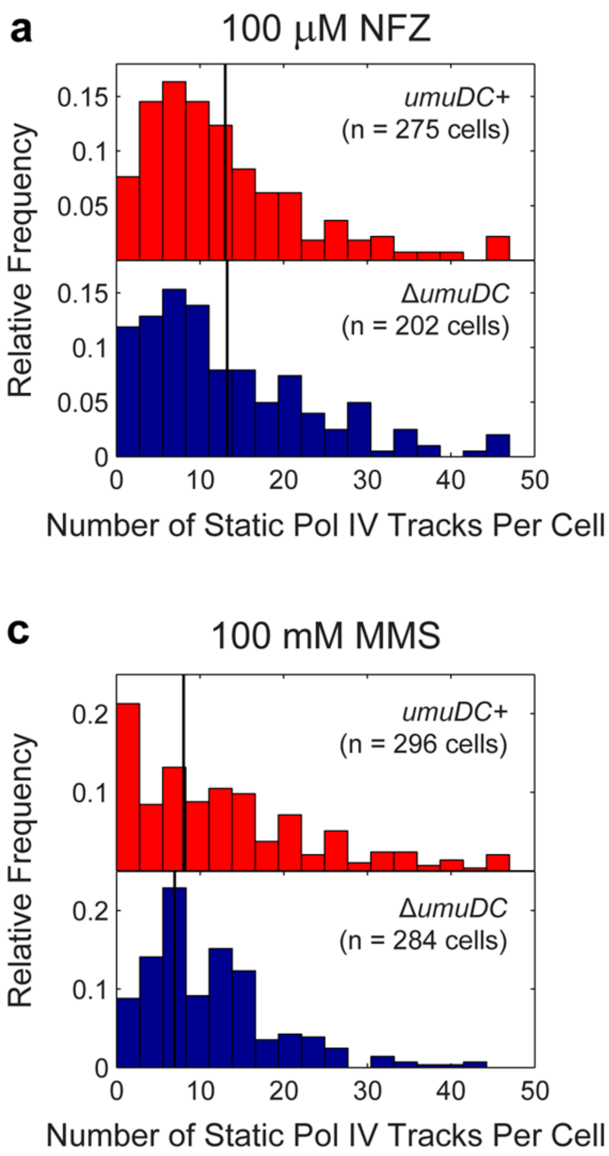

Supplementary Figure 16. Effect of UmuD on Pol IV localization in NFZ- and MMS-treated cells. Distributions of the number of static Pol IV-PAmCherry tracks per cell in cells treated with 100 μM NFZ (a) or 100 mM MMS (c) in the *umuDC*$^{+}$ and Δ*umuDC* backgrounds. The mean of each distribution is indicated by a solid line. Distributions of the long-axis cellular localization of static Pol IV-PAmCherry tracks in cells treated with 100 μM NFZ (b) or 100 mM MMS (d) in the *umuDC*$^{+}$ and Δ*umuDC* backgrounds. The data in the top panels of (a) and (c) are repeated from Fig. 4a, the data in the top panels of (b) are repeated from Fig. 2c, and the data in the top panels of (d) are repeated from Fig. 2e to enable comparison.

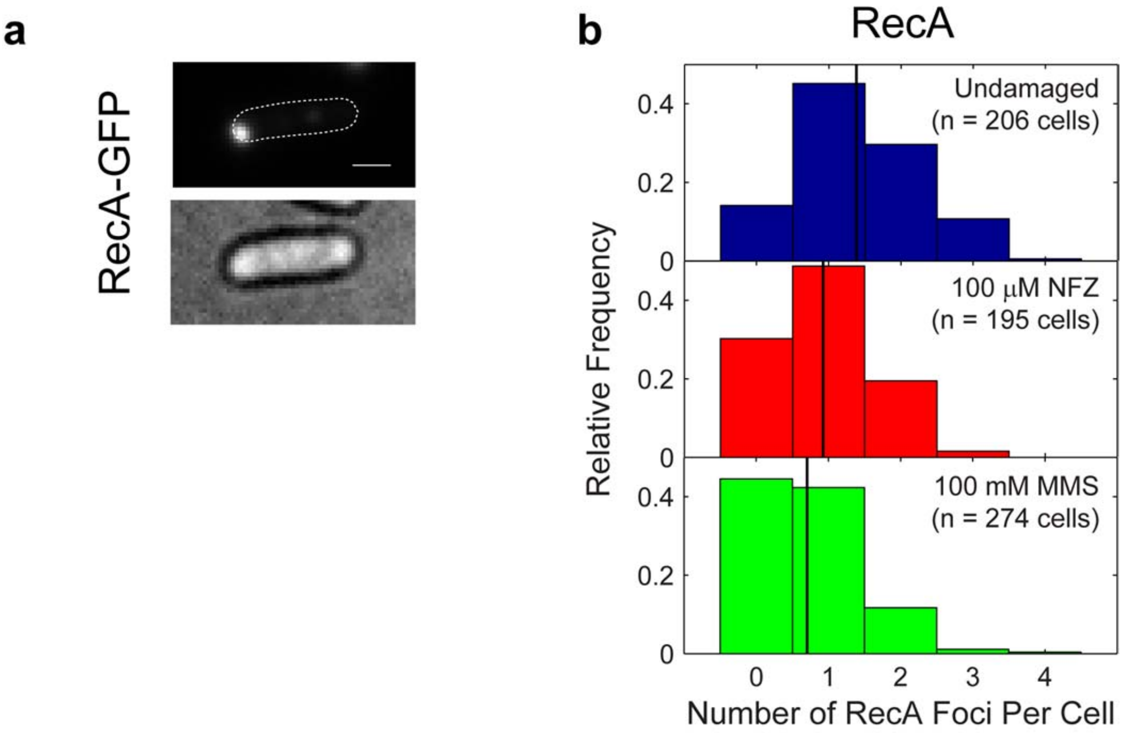

Supplementary Figure 17. Additional data for RecA-GFP imaging. (a) Top panel: representative fluorescence micrograph of RecA-GFP foci with an overlay of the cell outline. Bottom panel: the corresponding brightfield micrograph. (Scale bars: 1 μm.) (b) Distributions of the number of RecA-GFP foci per cell in undamaged (top), 100 μM NFZ-treated (middle), and 100 mM MMS-treated (bottom) cells. The mean of each distribution is indicated by a solid line.

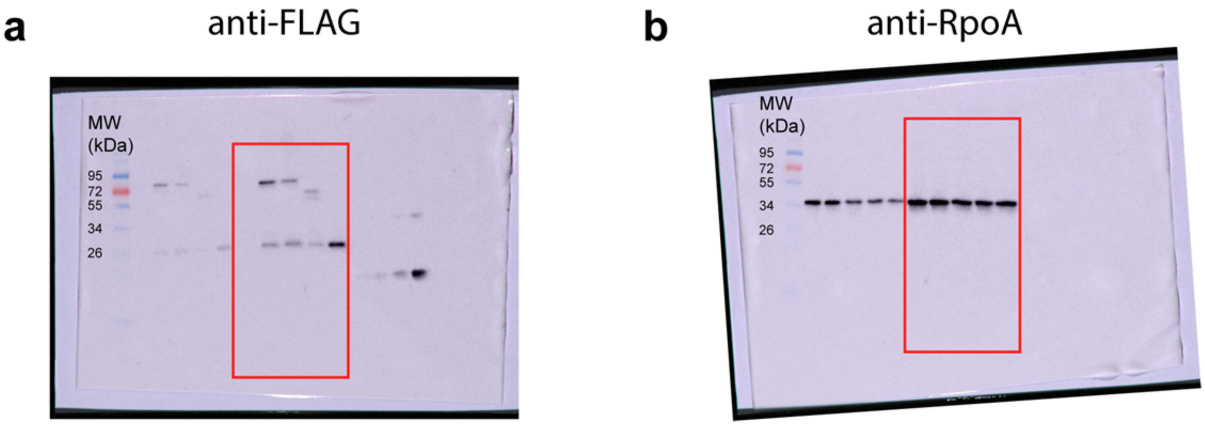

Supplementary Figure 18. Uncropped images of Western blots with size marker overlay for Supplementary Fig. 4a. Western blot analysis of strains containing Pol IV-PAmCherry (lanes 2 and 7), Pol IV-PAmCherry-FLAG (lanes 3 and 8: undamaged; lanes 4 and 9: treated with 100 μM NFZ), Pol IV^{CD}-PAmCherry-FLAG (lanes 5 and 10), and Pol IV^{LF}-PAmCherry-FLAG (lanes 6 and 11) constructs, and different concentrations of purified His_{10}-SUMO-FLAG (lanes 12 – 15) protein as a positive control. The protein ladder is in lane 1. Lanes 7 – 11 contain 3× the amount of lysate as matching samples in lanes 2 – 6. Lanes 12 – 15 contain 2, 4, 8, and 16 ng of purified protein respectively. Blots were probed with an anti-FLAG antibody to look at Pol IV expression (a) or an anti-RpoA antibody as a loading control (b). Cropped regions shown in Supplementary Fig. 4a are indicated by red boxes.

SUPPLEMENTARY TABLES

Supplementary Table 1: Oligonucleotides used in this study

Number	Designation	Sequence (5′−3′)
oJK022	Lambda red P1	GTGTAGGCTGGAGCTGCTTC
oJK100	RimKO_for	GGAGCACGCCGGGGCGCGGCTGAATAAAGC
oJK101	RimKO_rev	GCTTTATTCAGCCGCGCCCCGGCGTGCTCC
oJK102	dinB-kan-forward	CTTTACCAGTGTTGAGAGGTGAGCAATGCGTAAAATCATTGTG TAGGCTGGAGCTGCTTC
oJK103	dinB-kan-reverse	ACCAGAATATACATAATAGTATACATCATAATCCCAGCACCAT ATGAATATCCTCCTTA
oJK152	pUC19-Sherratt-linker-rev	GAATTCGCCAGAACCAGCAGCGGAGCCAGCCGAGCCACCGCC ACCGCTGAGCT
oJK162	linker-ITA-for	ATTACACGTCTTGAGCGATTGAGCTCGGCTGGCTCCGCTGCTG
oJK163	mYpet-ITA-rev	GAAGCAGCTCCAGCCTACACTTATTTGTACAATTCATT
oJK165	pKD4-ITA-for	TACAAATAAGTGTAGGCTGGAGCTGCT
oJK166	pKD4-ITA-rev	CAGCAGCGGAGCCAGCCGAGCTCAATCGCTCAAGACGTGTAA T
oJK167	Ypet-Sherratt-linker-for	TCGGCTGGCTCCGCTGCTGGTTCTGGCGAATTCATGAGTAAAG GTGAAGAATTA
oJK195	SSB-Ypet_for	TGCAGCATTACACGTCTTGAGCGATTGATGGCCAGCAGAGGCG TA
oJK196	SSB-Ypet_rev	GCCAGAACCAGCAGCGGAGCCAGCCGAGCTGAACGGAATGTC ATCATC
oJK197	lacZ-SSB-mYpet-KI_for	TGTGGAATTGTGAGCGGATAACAATTTCACACAGGAAACAGCT ATGGCCAGCAGAGGCGTAAA
oJK198	lacZ-SSB-mYpet-KI_rev	TCATCATATTTAATCAGCGACTGATCCACCCAGTCCCAGACGA AGATGAATATCCTCCTTAGTTCCTA
oJK216	dnaQ-mCherry-for	CCCGTCTCGATCTGGTGCAGAAGAAAGGCGGAAGTTGCCTCTG GCGAGCATCGGCTGGCTCCGCTGC
oJK217	dnaQ-mCherry-rev	TGCTCGAAAAATCGCCCAAGTCGCTATTTTTAGCGCCTTTCAC AGGTATTCTTATGAATATCCTCCTTAG
oJK239	dinB-mYpet-rev-pur	CAGTGATACCCTCATAATAATGCACACCAGAATATACATAATA GTATACATCTTATGAATATCCTCCTTAGTTCC
oJK275	pKD4-ITA-DinB-rev	CATGAATGATTTTACGCATAATCGCTCAAGACGTGTAAT
oJK276	DinB-pKD4-ITA-for	TACACGTCTTGAGCGATTATGCGTAAAATCATTCATGTGG
oJK294	pKD4_9_30_G4S_rev	GCCACCGCCGCCAGAACCACCACCACCAGAACCACCACCACC AATCGCTCAAGACGTGTAATGC
oJK298	pKD4_G4S_PAmCherry_f or	GGTGGTGGTGGTTCTGGCGGCGGTGGCAGTGGTGGCGGTGGCA GTATGGTTAGCAAGGGCGAGGAG
oJK299	pKD4_G4S_PAFP_rev	ACTTCGAAGCAGCTCCAGCCTACACTTACTTGTACAGCTCGTC CATGC
oJK305	dinB-G4S-FP_KI_for	GTGACGTTGCTTGACCCGCAAATGGAAAGACAACTGGTGCTGG GATTAGGTGGTGGTGGTTCTGGT
oJK325	dinB::PAmCherry-for	TCTCAAACCCTGAAATCACTGTATACTTTACCAGTGTTGAGAG GTGAGCAATGGTTAGCAAGGGCGAG
oJK335	pKD4_DinB_PAmCherry	CACCGCCGCCAGAACCACCACCACCAGAACCACCACCACCTA ATCCCAGCACCAGTTGT
oJK336	pKD4_DinB_dC6_PAmCh erry	CACCGCCGCCAGAACCACCACCACCAGAACCACCACCACCTCT TTCCATTTGCGGGTC
oJK343	DinB_cat-G4S-FP-FRT	GCTTTGGCAAATTTGGCCGCATTTTGTGGGAGCGTAGTCAGGG GATTGACGGTGGTGGTGGTTCTGGT
oJK344	dinB_700_718	GTTAACAGCGAACGGTTGC

oJK345	pKD4-PAm-FLAG-for	GGCAGCGACTACAAAGACGATGACGACAAGTAAGTGTAGGCT GGAGCT
oJK346	pKD4-PAm-FLAG-rev	TGTAGTCGCTGCCCTTGTACAGCTCGTCCATG
oJK353	LF-PAmCherry-KI-for	CCTGAAATCACTGTATACTTTACCAGTGTTGAGAGGTGAGCAA TGGTCGGCGTGGAACGCACG
SO114	dinB-F13V-For	GTGGATATGGACTGCTTTGTCGCCGCAGTGGAGATGCGC
SO115	dinB-F13V-Rev	GCGCATCTCCACTGCGGCGACAAAGCAGTCCATATCCAC
SO116	D103N_for	TGAACCGTTGTCACTGAATGAGGCTTATCTCGATGTC
SO117	D103N_rev	GACATCGAGATAAGCCTCATTCAGTGACAACGGTTCA
SO251	DinB-pKD4-ITA-rev	GTATAGGAACTTCGAAGCAGCTCCAGCCTACACTCATAATCCC AGCACCAGTTG
SO252	dinB_-20-+20-for	CCAGTGTTGAGAGGTGAGCAATGCGTAAAATCATTCATGT
SO278	rc_pKD4_9_30	AATCGCTCAAGACGTGTAATGC

Supplementary Table 2: Plasmids used in this study

#	Designation	Containing Strain	Source or Reference
1	pET11T-DinB	JEK1	pDFJ1[1]
2	pKD3	JEK42	[2]
3	pKD4	JEK45	[2]
4	pET21a-SSB	JEK121	Gift of Timothy Lohman, Washington University School of Medicine
5	pSIM5	JEK168	[3]
6	pCP20	JEK169	[4]
7	pKD46	JEK170	[2]
8	pUC19-mYPet	JEK296	Gift of Johan Paulsson, Harvard Medical School (containing strain: DHL1731)
9	pUC19-linker-mYPet	JEK361	This study
10	pKD4-linker-mYPet	JEK366	This study
11	pKD4-SSB-linker-mYPet	JEK372	This study
12	pKD3-linker-mYPet	JEK425	This study
13	pBAD-HisB-PAmCherry1	JEK493	Gift of Vladislav Verkhusha, Albert Einstein College of Medicine (Addgene #31931)
14	pET3a-mMaple3	JEK539	[5]
15	pKD4-G4S-PAmCherry	JEK573	This study
16	pKD4-G4S-mMaple3	JEK574	This study
17	pKD4-DinB-F13V-PAmCherry	JEK698	This study
18	pKD4-DinB-rim-dC6-PAmCherry	JEK699	This study
19	pKD4-DinB-D103N-PAmCherry	JEK707	This study
20	pKD4-G4S-PAmCherry-FLAG	JEK758	This study
21	pKD4-DinB-G4S-PAmCherry-FLAG	ET190	This study
22	pASK-IBA3plus-HupA-EYFP	ET209	[6]
23	pKD4-DinB	—	This study
24	pKD4-DinB-RimMut	—	This study
25	pKD4-DinB-F13V	—	This study
26	pKD4-DinB-D103N	—	This study

Supplementary Table 3: *Escherichia coli* bacterial strains used in this study

Strain	Designation or description	Relevant genotype	Construction or source strain designation	Reference
JEK11	Δ*umuDC*	Δ(*umuDC)595::cat*	JJF379 (Gift of Graham Walker, Massachusetts Institute of Technology)	[7]
JEK168	*E.coli* K-12 type strain with Cm^R recombineering plasmid	pSIM5 in MG1655	Transformation: pSIM5 → JEK5	[3]
JEK170	*E.coli* K-12 type strain with Amp^R recombineering plasmid	pKD46 in MG1655	Transformation: pKD46 → JEK5	[2]
JEK174	*E. coli* λ Red recombineering strain	*Δ(argF-lac)U169 mutS<>cat [lambda cI857 Δ(cro-bioA)]*	CH1358 (Gift of Diarmaid Hughes, Uppsala University)	[8]
JEK274/ JEK337	Δ*dinB*	MG1655 Δ*dinB::frt-kan-frt*	λ Red: dinB::FRT-Kan-FRT → MG1655 pKD46	This study
JEK395	SSB-mYPet	MG1655 *lacZ::ssb-mypet-frt-kan-frt*	λ Red: SSB-mYPet-FRT-Kan-FRT → MG1655 pSIM5	This study
JEK414	*$lexA^+$*	*$lexA^+$ rpsL31 xyl-5 mtl-1 galK2 lacY1 tsx-33 supE44 thi-1 hisG4[Oc] argE3[Oc] araD139 thr-1 Δ[gpt-proA]62 sulA211*	RW118	[9]
JEK418	Δ*lexA*	*lexA51(Def) rpsL31 xyl-5 mtl-1 galK2 lacY1 tsx-33 supE44 thi-1 hisG4[Oc] argE3[Oc] araD139 thr-1 Δ[gpt-proA]62 sulA211*	RW542	[10]
JEK466	ε-mYPet	CH1358 *dnaQ-mypet-frt-cat-frt*	λ Red: mYPet-FRT-Cm-FRT → CH1358	This study
JEK594/ JEK625	Pol IV-PAmCherry	MG1655 *dinB-PAmCherry-frt-kan-frt*	λ Red: PAmCherry-FRT-Kan-FRT → MG1655 pSIM5	This study
JEK596/ JEK626	Pol IV-mMaple3	MG1655 *dinB-mMaple3-frt-kan-frt*	λ Red: mMaple3-FRT-Kan-FRT → MG1655 pSIM5	This study
JEK599	Pol I-PAmCherry	AB1157 *polA-PAmCherry-frt-kan-frt*	RRL361	[11]
JEK600	ε-PAmCherry	AB1157 *dnaQ-PAmCherry-frt-kan-frt*	RRL508	[11]
JEK680	Δ*lexA* Pol IV-PAmCherry	RW542 *dinB-PAmCherry-frt-kan-frt*	P1*vir*: JEK594 → JEK418	This study
JEK713	Δ*dinB*::PAmCherry	MG1655 *dinB::PAmCherry-frt-kan-frt*	λ Red: dinB::PAmCherry-FRT-Kan-FRT → MG1655 pSIM5	This study
JEK717/ JEK726	Pol $IV^{R,C}$-PAmCherry	MG1655 *$dinB^{R,C}$-PAmCherry-frt-kan-frt*	λ Red: Pol $IV^{R,C}$-PAmCherry-FRT-Kan-FRT → MG1655 pSIM5	This study
JEK719	Pol IV^{CD}-PAmCherry	MG1655 *$dinB^{CD}$-PAmCherry-frt-kan-frt*	λ Red: $dinB^{LF}$::PAmCherry-FRT-Kan-FRT → MG1655 pSIM5	This study

JEK739	Pol IV-D103N-PAmCherry	MG1655 *dinB-D103N-PAmCherry-frt-kan-frt*	λ Red: Pol IV-D103N-PAmCherry-FRT-Kan-FRT → MG1655 pSIM5	This study
JEK759	Δ*lexA* SSB-mYPet	RW542 *lacZ::ssb-mypet-frt-kan-frt*	P1*vir*: JEK395 → JEK418	This study
JEK762	Δ*lexA* SSB-mYPet	RW542 *lacZ::ssb-mypet-frt*	JEK759 pCP20 Flp-FRT recombination	This study
JEK763	*lexA*$^{+}$ SSB-mYPet	RW118 *lacZ::ssb-mypet-frt-kan-frt*	P1*vir*: JEK395 → JEK414	This study
JEK765	Pol IV-PAmCherry-FLAG	MG1655 *dinB-PAmCherry-flag-frt-kan-frt*	λ Red: PAmCherry-FLAG-FRT-Kan-FRT → MG1655 pSIM5	This study
JEK766	Δ*lexA* Pol IV-PAmCherry SSB-mYPet	RW542 *dinB-PAmCherry-frt-kan-frt lacZ::ssb-mypet-frt*	P1*vir*: JEK594 → JEK762	This study
JEK769	Δ*lexA* Pol IV^{CD}-PAmCherry SSB-mYPet	RW542 *dinB*^{CD}*-PAmCherry-frt-kan-frt lacZ::ssb-mypet-frt*	P1*vir*: JEK719 → JEK762	This study
JEK770	*lexA*$^{+}$ SSB-mYPet	RW118 *lacZ::ssb-mypet-frt*	JEK763 pCP20 Flp-FRT recombination	This study
JEK781	Δ*lexA* Pol IV-PAmCherry-FLAG SSB-mYPet	RW542 *dinB-PAmCherry-flag-frt-kan-frt lacZ::ssb-mypet-frt*	P1*vir*: JEK765 → JEK762	This study
JEK783	Δ*lexA* Pol IV-mMaple3 SSB-mYPet	RW542 *dinB-mMaple3-frt-kan-frt lacZ::ssb-mypet-frt*	P1*vir*: JEK596 → JEK762	This study
JEK784	*lexA*$^{+}$ Pol IV-PAmCherry SSB-mYPet	RW118 *dinB-PAmCherry-frt-kan-frt lacZ::ssb-mypet-frt*	P1*vir*: JEK594 → JEK770	This study
JEK789	Δ*lexA* Pol IV-PAmCherry ε-mYPet	RW542 *dinB-PAmCherry-frt-kan-frt dnaQ-mypet-frt-cat-frt*	P1*vir*: JEK466 → JEK680	This study
JEK790	Δ*lexA* Pol IV^{R,C}-PAmCherry SSB-mYPet	RW542 *dinB*^{R,C}*-PAmCherry-frt-kan-frt lacZ::ssb-mypet-frt*	P1*vir*: JEK717 → JEK762	This study
JEK791	Δ*lexA* Pol IV-D103N-PAmCherry SSB-mYPet	RW542 *dinB-D103N-PAmCherry-frt-kan-frt lacZ::ssb-mypet-frt*	P1*vir*: JEK739 → JEK762	This study
JEK796	Δ*lexA* Pol IV-PAmCherry	RW542 *dinB-PAmCherry-frt*	JEK680 pCP20 Flp-FRT recombination	This study
JEK800	Δ*lexA* ε-PAmCherry SSB-mYPet	RW542 *dnaQ-PAmCherry-frt-kan-frt lacZ::ssb-mypet-frt*	P1*vir*: JEK600 → JEK762	This study
JEK801	Pol IV^{LF}-PAmCherry	MG1655 *dinB*^{LF}*-PAmCherry-frt-kan-frt*	λ Red: Pol IV^{LF}-PAmCherry-FRT-Kan-FRT → MG1655 pSIM5	This study
JEK802	Δ*lexA* Pol IV^{LF}-PAmCherry SSB-mYPet	RW542 *dinB*^{LF}*-PAmCherry-frt-kan-frt lacZ::ssb-mypet-frt*	P1*vir*: JEK801 → JEK762	This study
ET166	RecA-GFP	*ygaD1::kan recAo1403 recA4136::gfp-901*	SS3041	[12]
ET213	Pol IV^{CD}-PAmCherry-FLAG	MG1655 *dinB*^{CD}*-PAmCherry-flag-frt-kan-frt*	λ Red: dinB^{LF}::PAmCherry-FLAG-FRT-Kan-FRT → MG1655 pSIM5	This study

ET217	Δ*lexA* HU-EYFP	pASK-IBA3plus/HupA-EYFP in RW542	Transformation: pASK-IBA3plus/HupA-EYFP → JEK418	This study
ET223	Δ*lexA* Pol IV^{CD}-PAmCherry-FLAG SSB-mYPet	RW542 *dinB^{CD}-PAmCherry-flag-frt-kan-frt lacZ::ssb-mypet-frt*	P1*vir*: ET213 → JEK762	This study
ET231	Pol IV^{LF}-PAmCherry-FLAG	MG1655 *dinB^{LF}-PAmCherry-flag-frt-kan-frt*	λ Red: Pol IV^{LF}-PAmCherry-FLAG-FRT-Kan-FRT → MG1655 pSIM5	This study
ET232	Δ*lexA* RecA-GFP	RW542 *ygaD1::kan recAo1403 recA4136::gfp-901*	P1*vir*: ET166 → JEK418	This study
ET236	Δ*lexA ΔumuDC* Pol IV-PAmCherry SSB-mYPet	RW542 Δ*umuDC595::cat dinB-PAmCherry-frt-kan-frt lacZ::ssb-mypet-frt*	P1*vir*: JEK11 → JEK766	This study
ET238	Δ*lexA* Pol IV^{LF}-PAmCherry-FLAG SSB-mYPet	RW542 *dinB^{LF}-PAmCherry-flag-frt-kan-frt lacZ::ssb-mypet-frt*	P1*vir*: ET231 → JEK762	This study
ET242	Δ*lexA* Pol I-PAmCherry SSB-mYPet	RW542 *polA-PAmCherry-frt-kan-frt lacZ::ssb-mypet-frt*	P1*vir*: JEK599 → JEK762	This study
ET244	Δ*lexA ΔdinB*::PAmCherry SSB-mYPet	RW542 *dinB::PAmCherry-frt-kan-frt lacZ::ssb-mypet-frt*	P1*vir*: JEK713 → JEK762	This study
ET248	YPet-β	AB1157 *kan-frt-kan-ypet-dnaN*	RRL190	[13]
ET252	Δ*lexA* Pol IV-PAmCherry YPet-β	RW542 *dinB-PAmCherry-frt frt-kan-frt-ypet-dnaN*	P1*vir*: ET248 → JEK796	This study
ET254	Δ*lexA* Pol IV-PAmCherry YPet-β	RW542 *dinB-PAmCherry-frt frt-ypet-dnaN*	ET252 pCP20 Flp-FRT recombination	This study
ET282	ParC-PAmCherry MukB-mYPet	AB1157 *parC-PAmCherry-frt-kan-frt mukB-mYPet-frt gyrA^{L83}-tet*	PZ147	[14]
ET286	HU-PAmCherry	BW25993 *hupA-PAmCherry-frt-cat-frt*	SX127	[5]
ET288	Δ*lexA* HU-PAmCherry SSB-mYPet	RW542 *hupA-PAmCherry-frt-cat-frt lacZ::ssb-mypet-frt*	P1*vir*: ET286 → JEK762	This study

Supplementary Table 4: Number of foci per cell for replisome markers SSB-mYPet, ε-mYPet, and YPet-β

**Number of Foci Per Cell (Mean ± s.e.m.) (p-value vs. Undamaged)**	**Undamaged**	**100 µM NFZ**	**100 mM MMS**
SSB-mYPet	1.47 ± 0.05	0.97 ± 0.05 ($p << 10^{-5}$)	1.32 ± 0.04 (NS)
ε-mYPet	2.05 ± 0.08	1.67 ± 0.09 ($p < 0.05$)	1.24 ± 0.02 ($p << 10^{-5}$)
YPet-β	1.72 ± 0.04	1.62 ± 0.04 ($p < 0.05$)	1.17 ± 0.03 ($p << 10^{-5}$)

Supplementary Table 5: Mean intensity of foci for replisome markers SSB-mYPet, ε-mYPet, and YPet-β

**Intensity* (arb. units) (Mean ± s.e.m.) (p-value vs. Undamaged)**	**Undamaged**	**100 µM NFZ**	**100 mM MMS**
SSB-mYPet	$6.6 \times 10^5 \pm 2 \times 10^4$	$1.59 \times 10^6 \pm 6 \times 10^4$ ($p << 10^{-5}$)	$6.3 \times 10^5 \pm 2 \times 10^4$ (NS)
ε-mYPet	$3.3 \times 10^5 \pm 1 \times 10^4$	$7.9 \times 10^5 \pm 4 \times 10^4$ ($p << 10^{-5}$)	$5.1 \times 10^5 \pm 1 \times 10^4$ ($p << 10^{-5}$)
YPet-β	$2.66 \times 10^5 \pm 7 \times 10^3$	$3.41 \times 10^5 \pm 8 \times 10^3$ ($p << 10^{-5}$)	$4.3 \times 10^5 \pm 1 \times 10^4$ ($p << 10^{-5}$)

*Note: different excitation powers were used for the different strains, so intensity values can only be compared between different treatment conditions for a single strain.

Supplementary Table 6: Mean number of static tracks per cell for Pol IV WT and mutants

**Number of Static Tracks (Mean ± s.e.m.) (p-value vs. WT)**	**Undamaged**	**100 µM NFZ**	**100 mM MMS**
Pol IV^{WT}	7.4 ± 0.3	13.0 ± 0.6	8.1 ± 0.5
Pol $IV^{R,C}$	4.8 ± 0.2 ($p << 10^{-5}$)	13.4 ± 0.8 (NS)	5.1 ± 0.2 ($p < 10^{-5}$)
Pol IV^{CD}	4.4 ± 0.4 ($p < 10^{-5}$)	13.0 ± 0.7 (NS)	—
Pol IV^{LF}*	2.8 ± 0.3 ($p << 10^{-5}$)	3.3 ± 0.2 ($p << 10^{-5}$)	—
Pol IV^{D103N}	7.5 ± 0.8 ($0.01 < p < 0.05$)	13.3 ± 0.6 (NS)	7.0 ± 0.3 (NS)

*Note: Western blot analysis (see Supplementary Note 5) indicates that Pol IV^{LF}-PAmCherry is likely cleaved in the cell.

Supplementary Table 7: Mean number of static tracks per cell for *umuDC*⁺ and Δ*umuDC*

**Number of Static Tracks (Mean ± s.e.m.) (p-value vs. *umuDC*⁺)**	**Undamaged**	**100 µM NFZ**	**100 mM MMS**
umuDC⁺	7.4 ± 0.3	13.0 ± 0.6	8.1 ± 0.5
Δ*umuDC*	6.1 ± 0.4 ($0.01 < p < 0.05$)	13.3 ± 0.7 (NS)	6.9 ± 0.3 (NS)

SUPPLEMENTARY NOTES

Supplementary Note 1: General plasmid and bacterial strain construction methods

Bacterial strains bearing fluorescent protein fusions to Pol IV and other proteins were constructed using lambda Red recombineering[2]. The *E. coli* K-12 type strain, MG1655, was transformed with the temperature-sensitive plasmid pSIM5, which contains the temperature-inducible phage recombinogenic Red operon. Following induction of the Red operon, the cells were electroporated in the presence of linear DNA fragments containing ~ 50 bp homology arms to the chromosomal site at which the fusion was to be introduced. Cells containing the inserted DNA fragment were selected by plating on LB agar plates containing the appropriate antibiotic and the pSIM5 plasmid was cured by growth at 37 °C. The ε-mYPet fusion was also constructed using lambda Red recombineering, but in strain CH1358 instead of pSIM5 in MG1655. CH1358, a *galK*$^{+}$ version of HME68[8], contains a temperature-inducible genomic copy of the lambda Red operon and a *mutS* mutation for more efficient recombineering. P1*vir* phage transduction was used to move all fusions into a fresh MG1655 isolate or into a different genetic background. The presence of the correct fusion was verified at each step by PCR amplification of genomic DNA and sequencing.

The plasmid pKD4 was modified by Gibson assembly[15] or site-directed mutagenesis to create templates for amplification of linear recombineering fragments. pKD4 contains a kanamycin resistance cassette (Kan^{R}) flanked by FRT sites, and fluorescent protein fusions were introduced upstream of this cassette. The related plasmid pKD3, containing a FRT-flanked chloramphenicol resistance cassette (Cm^{R}), was also used. Polymerase chain reaction (PCR) was performed using Q5 Hot Start Polymerase (New England Biolabs) or home-purified Pfu polymerase to generate recombineering fragments. When needed, the Kan^{R} cassette was removed by transforming cells with the temperature-sensitive helper plasmid pCP20, which expresses the Flp recombinase[4]. Following Flp-FRT recombination, a single FRT site remains in a scar of ~ 80 bp.

Fusions to Pol IV and ε were made by replacing the endogenous copy of the gene with an in-frame C-terminal fusion to a linker and fluorescent protein. The only cellular copy of SSB, however, cannot be replaced with a C-terminal fusion; cell viability requires the presence of two

SSB monomers with free C-terminal tails per SSB tetramer[16]. Therefore a second copy of SSB containing a C-terminal fluorescent protein fusion was introduced at the *lac* operon, similar to a previous description[13,17], replacing the first ~ 1700 nucleotides of the *lacZ* gene. Expression of the *lac* operon and thus this fusion was then induced by adding isopropyl β-D-1-thiogalactopyranoside (IPTG) to growth media. The SSB-mYPet fusion used the 12 amino acid linker SSAGSAAGSGEF. The ε-mYPet and ε-PAmCherry fusions used the similar 11 amino acid linker SAGSAAGSGEF. Pol IV function, however, was found to be impaired with shorter linkers; instead, the 20 amino acid linker $(GGGGS)_4$ was used.

Fusions for PALM imaging were generated using the photoactivatable fluorescent proteins PAmCherry1[18] and mMaple3[5]. For orthogonal two-color imaging, fusions were made using green fluorescent protein (GFP) or yellow fluorescent protein (YFP) variants. SSB and ε fusions were constructed with mYPet, a variant of YPet[19] containing the monomeric A206K mutation. The previously reported HU-EYFP fusion[6] contains a version of EYFP with several minor mutations relative to the standard sequence, but with the wild-type A206 residue. The previously reported RecA-GFP fusion[12] contains the GFPmut2 variant with the monomeric A206T mutation. The previously reported YPet-β fusion[13] contains the standard YPet sequence without the monomeric A206K mutation. We found that a Pol IV fusion to mYPet was significantly more sensitive to nitrofurazone (NFZ) than the corresponding mMaple3 fusion, which itself was more sensitive than the PAmCherry fusion (Supplementary Fig. 1a). Consistent with a previous report[20], we observed that NFZ sensitivity increased with a reduced linker length.

Supplementary Note 2: Detailed strain construction information

JEK274/JEK337: MG1655 Δ*dinB*. A recombineering fragment was generated by amplifying pKD4 with primers dinB-kan-forward and dinB-kan-reverse and transformed into JEK170 (MG1655 pKD46), then pKD46 was cured to give strain JEK274. P1*vir* transduction was used to transfer the Δ*dinB::frt-kan-frt* allele from strain JEK274 to a fresh JEK5 isolate to give strain JEK337.

JEK395: MG1655 SSB-mYPet. The linker sequence SSAGSAAGSGEF was inserted into plasmid pUC19-mYPet using site-directed mutagenesis with the primers Ypet-Sherratt-linker-for and pUC19-Sherratt-linker-rev to make plasmid pUC19-linker-mYPet. The linker and *mypet* gene were amplified from the resulting plasmid using the primers linker-ITA-for and mYpet-ITA-rev. The backbone of plasmid pKD4 was amplified using primers pKD4-ITA-for and pKD4-ITA-rev. The linker-mYPet fragment was inserted into the pKD4 backbone by Gibson assembly to make plasmid pKD4-linker-mYPet. This plasmid was digested with SacI, cleaving a restriction site at the 5′ terminus of the linker, then gel-purified. The *ssb* gene was amplified from plasmid pET21a-SSB using primers SSB-Ypet_for and SSB-Ypet_rev and combined with the linker-mYPet fragment by Gibson assembly to make plasmid pKD4-SSB-linker-mYPet. A recombineering fragment containing the full SSB-linker-mYPet-FRT-Kan-FRT sequence was amplified using primers lacZ-SSB-mYpet-KI_for and lacZ-SSB-mYpet-KI_rev and transformed into JEK168 (MG1655 pSIM5), then pSIM5 was cured to give strain JEK395.

JEK466: CH1358 ε-mYPet. A PCR product containing the linker sequence SAGSAAGSGEF and the *mypet* gene were amplified from the plasmid pUC19-linker-mYPet using the primers linker-ITA-for and mYpet-ITA-rev and combined via Gibson assembly with a backbone fragment of the plasmid pKD3, amplified by inverse PCR using the oligonucleotides pKD4-ITA-for and pKD4-ITA-rev, generating the plasmid pKD3-linker-mYPet. A recombineering fragment containing mYPet and the FRT-Cm^R-FRT cassette was amplified from the vector pKD3-linker-mYPet with the oligonucleotides dnaQ-mCherry-for and dnaQ-mCherry-rev and transformed into strain JEK174 (CH1358). The oligonucleotide dnaQ-mCherry-rev was later found to have two mismatches in the downstream flanking region, but sequencing revealed that these mismatches were not present in the final strain.

JEK594/JEK625: MG1655 Pol IV-PAmCherry. The *pamcherry1* gene was amplified from pBAD-HisB-PAmCherry1 (Addgene #31931) using primers pKD4_G4S_PAmCherry_for and pKD4_G4S_PAmCherry_rev. The backbone of plasmid pKD4 was amplified using primers pKD4_9_30_G4S_rev and pKD4_31_48_PAmCherry_for. The *pamcherry1* gene, with an added $(GGGGS)_4$ linker, was inserted into the pKD4 backbone by Gibson assembly to make plasmid pKD4-G4S-PAmCherry. A recombineering fragment was generated by amplifying pKD4-G4S-

PAmCherry with primers dinB-G4S-FP_KI_for and dinB-FP_KI_rev and transformed into JEK168 (MG1655 pSIM5), then pSIM5 was cured to give strain JEK594. P1*vir* transduction was used to transfer the *dinB-PAmCherry* allele from strain JEK594 to a fresh JEK5 isolate to give strain JEK625.

JEK596/JEK626: MG1655 Pol IV-mMaple3. The *mmaple3* gene was amplified from pET3a-mMaple3[5] using primers pKD4_G4S_PAmCherry_for and pKD4_G4S_PAFP_rev. The backbone of plasmid pKD4 was amplified using primers Lambda red P1 and pKD4_9_30_G4S_rev. The *mmaple3* gene was inserted into the pKD4 backbone by Gibson assembly to make plasmid pKD4-G4S-mMaple3. A recombineering fragment was generated by amplifying pKD4-G4S-mMaple3 with primers dinB-G4S-FP_KI_for and dinB-mYpet-rev-pur and transformed into JEK168 (MG1655 pSIM5), then pSIM5 was cured to give strain JEK596. P1*vir* transduction was used to transfer the *dinB-mMaple3* allele from strain JEK596 into a fresh JEK5 isolate to give strain JEK626.

JEK680: P1*vir* transduction was used to transfer the *dinB-PAmCherry* allele from strain JEK594 to strain JEK418.

JEK713: MG1655 Δ*dinB*::PAmCherry. A recombineering fragment was generated by amplifying pKD4-G4S-PAmCherry with primers dinB::PAmCherry-for and dinB-mYpet-rev-pur and transformed into JEK168 (MG1655 pSIM5), then pSIM5 was cured to give strain JEK713.

JEK717/JEK726: MG1655 Pol IV^{R,C}-PAmCherry. This strain bears the ^{303}VWP^{305} → ^{303}AGA^{305} mutation, eliminating the Pol IV-β rim contact, and a deletion of the six C-terminal residues (^{346}QLVLGL^{351}), eliminating the Pol IV-β cleft contact. The backbone of plasmid pKD4 was amplified using oligonucleotides Lambda red P1 and pKD4-ITA-DinB-rev. The *dinB* gene was amplified from plasmid pET11T-DinB using oligonucleotides DinB-pKD4-ITA-for and DinB-pKD4-ITA-rev and combined with the pKD4 backbone fragment using Gibson assembly, generating the plasmid pKD4-DinB. The plasmid pKD4-DinB-RimMut, containing the Pol IV gene with the mutation ^{303}VWP^{305} → AGA upstream of the FRT-Kan^{R}-FRT cassette, was generated from pKD4-DinB by site-directed mutagenesis. Briefly, the plasmid pKD4-DinB was

amplified using oligonucleotides RimKO_for and RimKO_rev, treated with DpnI, and transformed into *E. coli* DH5α λpir. This plasmid was then amplified by inverse PCR with the oligonucleotides Lambda red P1 and pKD4_DinB_dC6_PAmCherry. The backbone PCR fragment was combined by Gibson assembly with a second fragment containing PAmCherry, amplified from pBAD-PAmCherry using oligonucleotides pKD4_G4S_PAmCherry_for and pKD4_G4S_PAFP_rev, generating the plasmid pKD4-DinB-rim-dC6-PAmCherry, which contains both the ^{303}VWP^{305} → AGA mutation and a truncation of the final six Pol IV residues. A recombineering fragment containing a section of the *dinB* gene including these mutations, PAmCherry, and the FRT-Kan^{R}-FRT cassette was amplified with primers dinB_700_718 and dinB-mYpet-rev-pur and transformed into JEK168 (MG1655 pSIM5), then pSIM5 was cured to give JEK717. P1*vir* transduction was used to transfer the *dinB*^{R,C}*-PAmCherry* allele from strain JEK717 into a fresh JEK5 isolate to give strain JEK726.

JEK719: MG1655 Pol IV^{CD}-PAmCherry. A recombineering fragment was generated by amplifying pKD4-G4S-PAmCherry with primers DinB_cat-G4S-FP-FRT and dinB-mYpet-rev-pur and transformed into JEK168 (MG1655 pSIM5), then pSIM5 was cured to give strain JEK719.

JEK739: MG1655 Pol IV-D103N-PAmCherry. The plasmid pKD4-DinB-D103N, containing the Pol IV gene with the mutation D103N, was generated from pKD4-DinB using site-directed mutagenesis. Briefly, the plasmid pKD4-DinB was amplified using oligonucleotides D103N_for and D103N_rev, treated with DpnI, and transformed into *E. coli* DH5α λpir. The resulting plasmid was then amplified by inverse PCR with the oligonucleotides Lambda red P1 and pKD4_DinB_PAmCherry. The backbone PCR fragment was combined by Gibson assembly with a second fragment containing PAmCherry, amplified from pBAD-PAmCherry using oligonucleotides pKD4_G4S_PAmCherry_for and pKD4_G4S_PAFP_rev, generating the plasmid pKD4-DinB-D103N-PAmCherry. A recombineering fragment containing the entire *dinB* allele, PAmCherry, and the FRT-Kan^{R}-FRT cassette was amplified with primers dinB_-20-+20-for and dinB-mYpet-rev-pur and transformed into JEK168 (MG1655 pSIM5), then pSIM5 was cured to give JEK739.

JEK762: Δ*lexA* SSB-mYPet. P1*vir* transduction was used to transfer the *lacZ::ssb-mypet* allele from strain JEK395 to strain JEK418 to give strain JEK759. Plasmid pCP20 was then transformed into strain JEK759 and Flp-FRT recombination was used to remove the Kan cassette, giving strain JEK762.

JEK765: MG1655 Pol IV-PAmCherry-FLAG. Site-directed mutagenesis was used to make the plasmid pKD4-G4S-PAmCherry-FLAG. Briefly, the plasmid pKD4-G4S-PAmCherry was amplified using the oligonucleotides pKD4-PAm-FLAG-for and pKD4-PAm-FLAG-rev, treated with DpnI, and transformed into *E. coli* DH5α λpir. A recombineering fragment was generated by amplifying pKD4-G4S-PAmCherry-FLAG with primers dinB-G4S-FP_KI_for and dinB-mYpet-rev-pur and transformed into JEK168 (MG1655 pSIM5), then pSIM5 was cured to give strain JEK765.

JEK766: Δ*lexA* Pol IV-PAmCherry SSB-mYPet. P1*vir* transduction was used to transfer the *dinB-PAmCherry* allele from strain JEK594 to strain JEK762.

JEK769: Δ*lexA* Pol IV^{CD}-PAmCherry SSB-mYPet. P1*vir* transduction was used to transfer the *dinB*^{CD}*-PAmCherry* allele from strain JEK719 to strain JEK762.

JEK770: *lexA*$^{+}$ SSB-mYPet. P1*vir* transduction was used to transfer the *lacZ::ssb-mypet* allele from strain JEK395 to strain JEK414 to give strain JEK763. Plasmid pCP20 was then transformed into strain JEK763 and Flp-FRT recombination was used to remove the Kan cassette, giving strain JEK770.

JEK781: Δ*lexA* Pol IV-PAmCherry-FLAG SSB-mYPet. P1*vir* transduction was used to transfer the *dinB-PAmCherry-flag* allele from strain JEK765 to strain JEK762.

JEK783: Δ*lexA* Pol IV-mMaple3 SSB-mYPet. P1*vir* transduction was used to transfer the *dinB-mMaple3* allele from strain JEK596 to strain JEK762.

JEK784: *lexA*$^{+}$ Pol IV-PAmCherry SSB-mYPet. P1*vir* transduction was used to transfer the *dinB-PAmCherry* allele from strain JEK594 to strain JEK770.

JEK789: Δ*lexA* Pol IV-PAmCherry ε-mYPet. P1*vir* transduction was used to transfer the *dnaQ-mYPet* allele from strain JEK466 to strain JEK680.

JEK790: Δ*lexA* Pol IV^{R,C}-PAmCherry SSB-mYPet. P1*vir* transduction was used to transfer the *dinB*^{R,C}*-PAmCherry* allele from strain JEK717 to strain JEK762.

JEK791: Δ*lexA* Pol IV-D103N-PAmCherry SSB-mYPet. P1*vir* transduction was used to transfer the *dinB-D103N-PAmCherry* allele from strain JEK739 to strain JEK762.

JEK800: Δ*lexA* ε-PAmCherry SSB-mYPet. P1*vir* transduction was used to transfer the *dnaQ-PAmCherry* allele, containing the linker SAGSAAGSGEF, from strain JEK600 to strain JEK762.

JEK801: MG1655 Pol IV^{LF}-PAmCherry. The plasmid pKD4-DinB-F13V, containing the Pol IV gene with the mutation F13V, was generated from pKD4-DinB by site-directed mutagenesis. Briefly, the plasmid pKD4-DinB was amplified using oligonucleotides dinB-F13V-For and dinB-F13V-Rev, treated with DpnI, and transformed into *E. coli* DH5α λpir. The resulting plasmid was then amplified by inverse PCR with the oligonucleotides Lambda red P1 and pKD4_DinB_PAmCherry. The backbone PCR fragment was combined by Gibson assembly with a second fragment containing PAmCherry, amplified from pBAD-PAmCherry using oligonucleotides pKD4_G4S_PAmCherry_for and pKD4_G4S_PAFP_rev, generating the plasmid pKD4-DinB-F13V-PAmCherry. A recombineering fragment containing the little finger (LF) domain of Pol IV, PAmCherry, and the FRT-Kan^{R}-FRT cassette was amplified from plasmid pKD4-DinB-F13V-PAmCherry with primers LF-PAmCherry-KI-for and dinB-mYpet-rev-pur and transformed into JEK168 (MG1655 pSIM5), then pSIM5 was cured to give JEK801. Note that the F13V mutation is within the catalytic domain and is therefore not present in the Pol IV^{LF} fragment.

JEK802: Δ*lexA* Pol IV^{LF}-PAmCherry SSB-mYPet. P1*vir* transduction was used to transfer the *dinB^{LF}-PAmCherry* allele from strain JEK801 to strain JEK762.

ET217: Δ*lexA* HU-EYFP. Plasmid pASK-IBA3plus-HupA-EYFP was transformed by electroporation into strain JEK418.

ET213: MG1655 Pol IV^{CD}-PAmCherry-FLAG. A recombineering fragment was generated by amplifying the plasmid pKD4-G4S-PAmCherry-FLAG with primers DinB_cat-G4S-FP-FRT and dinB-mYpet-rev-pur and transformed into JEK168 (MG1655 pSIM5), then pSIM5 was cured to give strain ET213.

ET223: Δ*lexA* Pol IV^{CD}-PAmCherry-FLAG SSB-mYPet. P1*vir* transduction was used to transfer the *dinB^{CD}-PAmCherry-flag* allele from strain ET213 to strain JEK762.

ET231: MG1655 Pol IV^{LF}-PAmCherry-FLAG. The *dinB* gene was amplified from plasmid pKD4-DinB using oligonucleotides DinB-pKD4-ITA-for and pKD4_DinB_PAmCherry. The plasmid pKD4-G4S-PAmCherry-FLAG was amplified by inverse PCR with the oligonucleotides dinB-G4S-FP_KI_for and pKD4-ITA-DinB-rev. This backbone fragment was combined with the *dinB* gene by Gibson assembly to generate plasmid pKD4-DinB-G4S-PAmCherry-FLAG. A recombineering fragment was amplified from this plasmid with primers LF-PAmCherry-KI-for and dinB-mYpet-rev-pur and transformed into JEK168 (MG1655 pSIM5), then pSIM5 was cured to give ET231.

ET232: Δ*lexA* RecA-GFP. P1*vir* transduction was used to transfer the *recAo1403 recA-gfp* allele from strain ET166 to strain JEK418.

ET236: Δ*lexA ΔumuDC* Pol IV-PAmCherry SSB-mYPet. P1*vir* transduction was used to transfer the Δ*umuDC595::cat* allele from strain JEK11 to strain JEK762.

ET238: Δ*lexA* Pol IV^{CD}-PAmCherry-FLAG SSB-mYPet. P1*vir* transduction was used to transfer the *dinB^{LF}-PAmCherry-flag* allele from strain ET231 to strain JEK762.

ET242: Δ*lexA* Pol I-PAmCherry SSB-mYPet. P1*vir* transduction was used to transfer the *polA-PAmCherry* allele from strain JEK599 to strain JEK762.

ET244: Δ*lexA ΔdinB*::PAmCherry SSB-mYPet. P1*vir* transduction was used to transfer the *dinB::PAmCherry* allele from strain JEK713 to strain JEK762.

ET254: Δ*lexA* Pol IV-PAmCherry YPet-β. Flp-FRT recombination was used to remove the Kan cassette from strain JEK680, giving strain JEK796. Then P1*vir* transduction was used to transfer the *frt-kan-frt-ypet-dnaN* allele from strain ET248 to strain JEK796 to create strain ET252. Finally, Flp-FRT recombination was used to remove the Kan cassette, giving strain ET254.

ET288: Δ*lexA* HU-PAmCherry SSB-mYPet. P1*vir* transduction was used to transfer the *hupA-frt-cat-frt* allele from strain ET286 to strain JEK762.

Supplementary Note 3: Growth rates

Growth curves were measured in supplemented M9 glucose media plus IPTG following the same procedure as for imaging culture growth. The values below represent the mean and standard deviation from three independent experiments.

**Strain**	**Doubling Time (min) (Mean ± std. dev.)**
JEK414 (*lexA*$^{+}$)	41.5 ± 1.3
JEK418 (*ΔlexA*)	45.9 ± 1.1
JEK766 (*ΔlexA dinB-PAmCherry-frt-kan-frt lacZ::ssb-mypet-frt*)	44.1 ± 0.4

Supplementary Note 4: NFZ survival assay

Liquid cultures were treated with 40 μM or 100 μM NFZ or mock treated with DMF solvent as described in the Methods section. The number of colony forming units (CFUs) was

assessed before and after treatment and the fold change was calculated. The values below represent the mean and standard deviation from three biological replicates.

Strain	Fold Change in CFUs (Mean ± std. dev.)		
	**Mock**	**40 μM NFZ**	**100 μM NFZ**
JEK762 (*ΔlexA lacZ::ssb-mypet-frt*)	2.30 ± 0.39	Not determined	0.56 ± 0.06
JEK766 (*ΔlexA dinB-PAmCherry-frt-kan-frt lacZ::ssb-mypet-frt*)	2.30 ± 0.15	1.23 ± 0.22	0.60 ± 0.11

Supplementary Note 5: Western blotting

Western blot analysis of whole cell lysates was used to verify the expression level of the WT Pol IV-PAmCherry fusion protein, both undamaged and after NFZ treatment, and the corresponding Pol IV^{CD} and Pol IV^{LF} truncation mutants. Because a previously described Pol IV antibody[21] was not sensitive enough to detect endogenous cellular levels, we constructed Pol IV^{WT}-, Pol IV^{CD}-, and Pol IV^{LF}-PAmCherry-FLAG fusions with a C-terminal FLAG tag (amino acid sequence GSDYKDDDDK) for detection with an anti-FLAG antibody.

Culture growth and treatment with 100 μM NFZ was performed following the normal procedures for imaging. When cultures reached $OD_{600nm} \approx 0.15$, they were either treated with NFZ and grown for an additional hour or harvested. Cells were pelleted by centrifugation at 7,197 × g for 5 min at 4 °C and the pellet was flash frozen in liquid nitrogen. The pellet was then resuspended in (40 × vol × OD_{600nm}) μL of lysis buffer (where vol was the remaining culture volume in mL) prepared fresh following a previous report[22]. The lysis buffer consisted of B-PER Bacterial Protein Extraction Reagent (Thermo Scientific #78248) supplemented with 50 μg mL^{-1} lysozyme (EMD #5950), a 1:2000 dilution of Benzonase nuclease (Novagen #70746), and a 1:25 dilution of the working concentration of EDTA-free protease inhibitor cocktail (Roche #04693159001) dissolved in water following the manufacturer's instructions. The samples were incubated on ice for 10 min and then stored at −20 °C. The cell lysate was diluted in homemade 6× SDS-PAGE sample loading buffer (375 mM Tris·HCl, 12% sodium dodecyl sulfate (SDS), and 48% glycerol brought to pH 6.8, then bromophenol blue added to a final concentration of

0.25%) boiled for 5 min, separated on a precast SDS-PAGE gel (Bio-Rad #4569036: Any kD Mini-PROTEAN TGX), and transferred to a polyvinylidene difluoride membrane (PerkinElmer #NEF1002001PK: PolyScreen PVDF Hybridization Transfer Membrane) at 100 V for 1 h at 4 °C. The membrane was washed in TBST (tris-buffered saline + 0.1% Tween 20) buffer and then blocked overnight in 10% milk TBST at 4 °C.

The membrane was probed with a 1:5,000 dilution of a rabbit anti-FLAG antibody raised against the antigen Ac-C(dPEG4)DYKDDDDK-OH (a gift of Johannes Walter, Harvard Medical School) for 1 h at room temperature, washed in TBST 4 times for 5 min each, incubated with a 1:20,000 dilution of goat anti-rabbit IgG-HRP (Jackson ImmunoResearch #111-035-003) for 1 h at room temperature, and then washed as before. The membrane was incubated with HyGLO chemiluminescent HRP antibody detection reagent (Denville Scientific #E2400) and imaged on an Amersham Imager 600. All antibody dilutions were made in 10% milk TBST. A second membrane was prepared identically at the same time and probed with an anti-RpoA antibody as a loading control. For this blot, a 1:10,000 dilution of mouse anti-RpoA primary antibody (NeoClone #W0003) and a 1:20,000 dilution of rabbit anti-mouse IgG-HRP secondary antibody (Jackson ImmunoResearch #315-035-003) were used. The washing and detection steps were performed as above.

No bands were detected using the anti-FLAG antibody in a strain bearing a Pol IV-PAmCherry fusion without the C-terminal FLAG tag, as expected (Supplementary Fig. 4a). The lower molecular weight band in the FLAG-tagged constructs may reflect a PAmCherry cleavage product. In the Pol IV^{LF}-PAmCherry-FLAG construct, it is the only species present, suggesting that this construct may be cleaved completely in the cell. Blotting with the anti-RpoA antibody as a loading control revealed similar levels of the RNA polymerase α subunit in all strains tested (Supplementary Fig. 4a).

Supplementary Note 6: Estimate of static fraction of Pol IV molecules in PALM imaging with long integration times

The mean number of static Pol IV tracks increased from 7.4 ± 0.3 (mean ± s.e.m) in undamaged cells to 9.8 ± 0.6 in 40 μM NFZ-treated cells to 13.0 ± 0.6 in 100 μM NFZ-treated

cells under matched imaging conditions with a long integration time of 250 ms ($p < 10^{-3}$ for undamaged vs. 40 μM NFZ and $p < 10^{-4}$ for 40 vs. 100 μM NFZ) (Fig. 4a). These values represent only a fraction of the total number of Pol IV-PAmCherry molecules in the cell, both because some PAmCherry molecules are immature or otherwise unable to be activated[5] and because our imaging conditions are not designed to activate all molecules. To estimate the fraction of bound molecules, we measured the number of static tracks in fixed cells. We found a mean value of 35 ± 1 (mean ± s.e.m.) static tracks per cell. To account for the effect of fixation on PAmCherry activation, we compared the number of Pol IV-PAmCherry tracks in undamaged and fixed cells using a short integration time of 13.3 ms. We found a 40% reduction in the number of tracks per cell, broadly consistent with a previously reported 20% reduction for a different PAmCherry fusion in *E. coli*. From this measurement, we estimate that there should be approximately 58 Pol IV-PAmCherry activations in undamaged cells, and thus that 13% and 22% of Pol IV molecules are static in undamaged and 100 μM NFZ-treated cells respectively.

SUPPLEMENTARY REFERENCES

1. Jarosz, D. F., Godoy, V. G., Delaney, J. C., Essigmann, J. M. & Walker, G. C. A single amino acid governs enhanced activity of DinB DNA polymerases on damaged templates. *Nature* **439,** 225–8 (2006).

2. Datsenko, K. A. & Wanner, B. L. One-step inactivation of chromosomal genes in Escherichia coli K-12 using PCR products. *Proc. Natl. Acad. Sci. U. S. A.* **97,** 6640–5 (2000).

3. Sharan, S. K., Thomason, L. C., Kuznetsov, S. G. & Court, D. L. Recombineering: a homologous recombination-based method of genetic engineering. *Nat. Protoc.* **4,** 206–223 (2009).

4. Cherepanov, P. P. & Wackernagel, W. Gene disruption in Escherichia coli: TcR and KmR cassettes with the option of Flp-catalyzed excision of the antibiotic-resistance determinant. *Gene* **158,** 9–14 (1995).

5. Wang, S., Moffitt, J. R., Dempsey, G. T., Xie, X. S. & Zhuang, X. Characterization and development of photoactivatable fluorescent proteins for single-molecule-based superresolution imaging. *Proc. Natl. Acad. Sci. U. S. A.* **111,** 8452–7 (2014).

6. Bakshi, S. *et al.* Nonperturbative imaging of nucleoid morphology in live bacterial cells during an antimicrobial peptide attack. *Appl. Environ. Microbiol.* **80,** 4977–4986 (2014).

7. Woodgate, R. Construction of a umuDC operon substitution mutation in Escherichia coli. *Mutat. Res.* **281,** 221–5 (1992).

8. Thomason, L. C., Costantino, N., Shaw, D. V. & Court, D. L. Multicopy plasmid modification with phage lambda Red recombineering. *Plasmid* **58,** 148–58 (2007).

9. Ho, C., Kulaeva, O. I., Levine, A. S. & Woodgate, R. A rapid method for cloning mutagenic DNA repair genes: isolation of umu-complementing genes from multidrug resistance plasmids R391, R446b, and R471a. *J. Bacteriol.* **175,** 5411–9 (1993).

10. Fernández De Henestrosa, A. R. *et al.* Identification of additional genes belonging to the LexA regulon in Escherichia coli. *Mol. Microbiol.* **35,** 1560–1572 (2000).

11. Uphoff, S., Reyes-Lamothe, R., Garza de Leon, F., Sherratt, D. J. & Kapanidis, A. N. Single-molecule DNA repair in live bacteria. *Proc. Natl. Acad. Sci. U. S. A.* **110,** 8063–8 (2013).

12. Renzette, N. *et al.* Localization of RecA in Escherichia coli K-12 using RecA-GFP. *Mol. Microbiol.* **57,** 1074–85 (2005).

13. Reyes-Lamothe, R., Sherratt, D. J. & Leake, M. C. Stoichiometry and architecture of active DNA replication machinery in Escherichia coli. *Science* **328,** 498–501 (2010).

14. Zawadzki, P. *et al.* The localization and action of Topoisomerase IV in Escherichia coli chromosome segregation is coordinated by the SMC complex, MukBEF. *Cell Rep.* **13,**

2587–96 (2015).

15. Gibson, D. G. *et al.* Enzymatic assembly of DNA molecules up to several hundred kilobases. *Nat. Methods* **6,** 343–345 (2009).

16. Antony, E. *et al.* Multiple C-terminal tails within a single E coli SSB homotetramer coordinate DNA replication and repair. *J. Mol. Biol.* **425,** 4802–4819 (2013).

17. Reyes-Lamothe, R., Possoz, C., Danilova, O. & Sherratt, D. J. Independent positioning and action of Escherichia coli replisomes in live cells. *Cell* **133,** 90–102 (2008).

18. Subach, F. V *et al.* Photoactivatable mCherry for high-resolution two-color fluorescence microscopy. *Nat. Methods* **6,** 153–9 (2009).

19. Nguyen, A. W. & Daugherty, P. S. Evolutionary optimization of fluorescent proteins for intracellular FRET. *Nat. Biotechnol.* **23,** 355–60 (2005).

20. Mallik, S., Popodi, E. M., Hanson, A. J. & Foster, P. L. Interactions and localization of Escherichia coli error-prone DNA polymerase IV after DNA damage. *J. Bacteriol.* **197,** 2792–809 (2015).

21. Cafarelli, T. M. *et al.* A single residue unique to DinB-like proteins limits formation of the polymerase IV multiprotein complex in Escherichia coli. *J. Bacteriol.* **195,** 1179–93 (2013).

22. Landgraf, D., Okumus, B., Chien, P., Baker, T. A. & Paulsson, J. Segregation of molecules at cell division reveals native protein localization. *Nat. Methods* **9,** 480–2 (2012).
